# Further Study on Chemical Constituents of *Parnassia wightiana* Wall: Four New Dihydro-β-agarofuran Sesquiterpene Polyesters

**DOI:** 10.3390/ijms16059119

**Published:** 2015-04-23

**Authors:** Zhao-Feng Gao, Bo-Hang Zhou, Jie-Yu Zhao, Fang-Jun Cao, Le Zhou, Hui-Ling Geng

**Affiliations:** College of Science, Northwest A&F University, Yangling 712100, China; E-Mails: gaozhaofeng1990@126.com (Z.-F.G.); zhoubohang@nwsuaf.edu.cn (B.-H.Z.); ymjr00@163.com (J.-Y.Z.); junjun198789@126.com (F.-J.C.)

**Keywords:** dihydro-β-agarofuran, sesquiterpene, *Parnassia wightiana*, sesquiterpene alkaloid

## Abstract

Four new (**1**–**4**), along with six known (**5**–**10**) dihydro-β-agarofuran sesquiterpene polyesters were isolated from the whole plants of *Parnassia wightiana*. The new compounds were structurally elucidated through spectroscopic analysis including UV (Ultraviolet Spectrum), IR (Infrared Spectrum), ^1^H-NMR (^1^Hydrogen-Nuclear Magnetic Resonance), ^13^C-NMR (^13^Carbon-Nuclear Magnetic Resonance), DEPT (Distortionless Enhancement by Polarization Transfer), ^1^H-^1^H COSY (^1^H-^1^H Correlation Spectroscopy), HSQC (Heteronuclear Single Quantum Coherence), HMBC (Heteronuclear Multiple Bond Correlation), NOESY (Nuclear Overhauser Enhancement Spectroscopy) and HR-MS (High Resolution Mass Specttrum) and their absolute configurations were proposed by comparison of NOESY spectra and specific optical rotations with those of known compounds and biosynthesis grounds. Compound **2** is the first sesquiterpene alkaloid isolated from this plant. New compounds **1**–**4** exhibited some cytotoxic activities against NB4, MKN-45 and MCF-7 cells at 20 μM and of which **4** showed the highest activity against NB4 and MKN-45 cells with inhibition rates of 85.6% and 30.5%, respectively.

## 1. Introduction

*Parnassia wightiana* wall (Saxifragaceae), commonly known as Ji-mei-hua-cao, Cang-er-qi, Qiao-mai-ye or Ding-chuang-cao in China, is a perennial herb and mainly distributes in Qinling Mountains in China. Its dried whole plants have been used as Chinese folk medicine for the treatment of leukorrhea, cough, haematemesis, carbuncle, irregular menstruation, hypertension, malaria, kidney stones, gall stones,* etc.* Recently, we reported eight dihydro-β-agarofuran sesquiterpene polyesters including five new compounds isolated from this plant and their anticancer activity [1]. In addition, some dihydro-β-agarofuran sesquiterpenes with antifeedant and antifungal activities were isolated from this plant as well [2,3]. Here we report four new along with six known β-dihydroagarofuran sesquiterpene polyesters have been obtained and the isolation and structural elucidation of these sesquiterpene esters.

## 2. Results and Discussion

**Figure 1:**
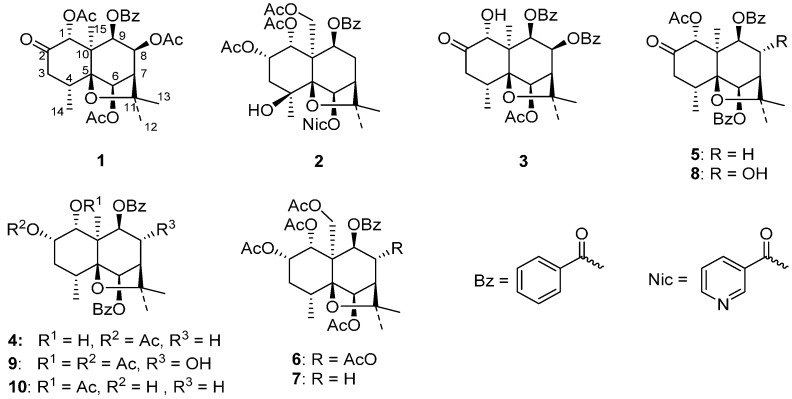
Structures of compounds **1**–**10** isolated from *P. wightiana*.

The ethyl acetate extract from the whole plants of *P. wightiana* was subjected to column chromatography on silica gel, preparative HPLC (High Performance Liquid Chromatography) and preparative silica gel TLC (Thin Layer Chromatography), affording four new dihydro-β-agarofuran sesquiterpenes (**1**–**4**), along with six known compounds (**5**–**10**) previously isolated from the same plant (Figure 1) [1].

Compound **1** was obtained as a pale yellow solid with [α]_D_^19^ = −8.661° (*c* = 0.26, CH_3_OH). The pseudo-molecular ions at* m*/*z*: 553.2035 [M + Na]^+^ in HR-ESI-MS (Figure S6) in conjunction with an analysis of ^1^H- and ^13^C-NMR spectra (Figures S1 and S2) afforded a molecular formula of C_28_H_34_O_10_. The IR showed the presence of carbonyl (1754, 1734 cm^−1^) and aromatic groups (3063, 1602 cm^−1^). The UV spectrum showed the presence of benzoate moieties with maximum absorption peaks at 204, 232, 274 and 282 nm.

The ^1^H- and ^13^C-NMR spectroscopic data of compound **1** (Table 1 and Table 2) showed the structural characteristics of sesquiterpene polyol esters with a dihydro-β-agarofuran skeleton found in the same plant [1]. The ^1^H-NMR displayed the presence of three acetyl groups (δ_H_ 1.60 (s, 3H), 1.78 (s, 3H), 2.05 (s, 3H)), three tertiary methyl groups (δ_H_ 1.22 (s, 3H), 1.45 (s, 3H), 1.58 (s, 3H)), one secondary methyl group (δ_H_ 0.90 (d, 7.6 Hz, 3H)) and one benzoate group (δ_H_ 7.39–7.92, 5H). Additionally, the ^13^C-NMR and DEPT (Distortionless Enhancement by Polarization Transfer) spectra revealed that **1** also contained one methylene group (δ_C_ 43.1 (CH_2_)), six methine groups with four linked to an oxygen atom (δ_C_ 38.1 (CH), 53.3 (CH), 69.0 (CH–O–), 72.6 (CH–O–), 76.0 (CH–O–), 77.2 (CH–O–)), three quaternary sp^3^ carbons with two bearing an oxygen atom (δ_C_ 54.0 (C), 84.0 (C–O–), 88.7(C–O–)), one ketone carbonyl group (δ_C_ 204.8) and four ester carbonyl groups (δ_C_ 165.4, 169.3, 169.5, 169.8). The complete assignments of all carbon and hydrogen signals were carried out by an analysis of ^1^H-^1^H COSY (^1^H-^1^H Correlation Spectroscopy), HSQC (Heteronuclear Single Quantum Coherence) (Figure S3), HMBC (Heteronuclear Multiple Bond Correlation) (Figure S4) and DEPT spectra (Table 1). The signals observed at δ_H_ 5.16 (d, *J* = 6.4 Hz), 5.35 (s), 5.53 (dd, *J* = 3.2, 6.4 Hz) and 5.82 (s) were assigned to H-9, H-6, H-8 and H-1, respectively.

**Table 1:** ^1^H-NMR data of compounds **1**–**4** (500 MHz; **1** and **2**: CD_3_OD; **3** and **4**: CD_3_Cl).

No.	1, δ_H_, *J* in Hz	2, δ_H_, *J* in Hz	3, δ_H_, *J* in Hz	4, δ_H_, *J* in Hz
1	5.82, s	5.54, d, 3.1	5.19, d, 4.5	4.62, dd, 4.0, 5.4
1-OH	–	–	3.19, d, 4.5	1.82, d, 5.5
2	–	5.41, m	–	5.34, q-like, 3.4
3_eq_	2.10, dd, 1.6, 12.9	1.90, dd, 2.5, 15.0,	2.37, br d, 13.1	1.89, br d, 13.0
3_ax_	3.15, dd, 7.3, 12.9	2.22, m	3.24, dd, 7.6, 13.1	2.41, 4 × d, 3.4, 6.2, 13.0
4	2.73, m	–	2.86, m	2.51, m
6	5.35, s	6.30, s	5.49, s	5.67, s
7	2.56, d, 3.2	2.33, br s	2.78, d, 2.9	2.42, d, 3.2
8_eq_	–	2.25, m	–	2.29, dd, 3.2, 16.4
8_ax_	5.53, dd, 3.2, 6.4	2.59, 4 × d, 3.5, 7.0, 16.0	5.84, dd, 3.2, 6.6	2.58, 4 × d, 3.2, 7.0, 16.4
9	5.16, d, 6.4	5.42, d, 7.0	5.44, d, 6.6	5.08, d, 7.0
12	1.45, s	1.49, s	1.52, s	1.46, s
13	1.58, s	1.53, s	1.82, s	1.50, s
14	0.90, d, 7.6	1.41, s	1.01, d, 7.6	1.21, d, 7.7
15	1.22, s	4.99, d, 13.0; 4.35, d, 13.0	1.22, s	1.42, s
AcO-1	1.60, s	1.47, s	–	–
AcO-2	–	1.99, s	–	2.02, s
AcO-6	2.05, s	–	2.15, s	–
AcO-8	1.78, s	–	–	–
AcO-15	–	2.22, s	–	–
2'/6'	7.92, d, 8.4	7.94, d, 7.8	8.04, d, 7.5	8.09, d, 8.4
3'/5'	7.39, t, 7.9	7.39, t, 7.7	7.44, t, 7.5	7.46, t, 7.6
4'	7.54, t, 7.4	7.54, t, 7.4	7.58, t, 7.5	7.57, t, 7.4
2''/6''	–	9.23, s (2''-H); 8.47, d, 8.0 (6''-H)	7.78, d, 7.5	8.07, d, 8.4
3''/5''	–	7.50, dd, 2.6, 8.0 (5''-H)	7.26, t, 7.8	7.50, d, 7.8
4''	–	8.68, br s (4''-H)	7.47, t, 7.5	7.61, t, 7.4

**Table 2:** ^13^C-NMR and DEPT (Distortionless Enhancement by Polarization Transfer) data of compounds **1**–**4** (125 MHz; **1** and **2**: CD_3_OD; **3** and **4**: CD_3_Cl).

No.	1	2	3	4
1	77.2, CH	70.9, CH	75.1, CH	68.9, CH
2	204.8, C	69.1, CH	211.5, C	73.9, CH
3	43.1, CH_2_	41.2, CH_2_	42.8, CH_2_	31.2, CH_2_
4	38.1, CH	69.7, C	38.4, CH	34.1, CH
5	88.7, C	90.8, C	88.7, C	90.0, C
6	76.0, CH	79.2, CH	77.3, CH	80.2, CH
7	53.3, CH	49.1, CH	53.7, CH	49.1, CH
8	69.0, CH	34.3, CH_2_	69.9, CH	31.6, CH_2_
9	72.6, CH	68.0, CH	72.8, CH	73.4, CH_2_
10	54.0, C	55.3, C	56.3, C	51.6, C
11	84.0, C	84.5, C	83.8, C	82.8, C
12	30.2, CH_3_	24.7, CH_3_	31.2, CH_3_	30.9, CH_3_
13	25.7, CH_3_	28.2, CH_3_	26.8, CH_3_	26.1, CH_3_
14	16.9, CH_3_	24.4, CH_3_	18.4, CH_3_	18.8, CH_3_
15	18.58 *, CH_3_	65.2, CH_2_	18.9, CH_3_	19.2, CH_3_
AcO-1	18.62 *, CH_3_; 169.5, C	19.1, CH_3_; 169.6, C	–	–
AcO-2	–	19.6, CH_3_; 170.0, C	–	21.5, CH_3_; 171.5, C
AcO-6	19.6, CH_3_; 169.8, CH_3_	–	21.2, CH_3_; 169.4, C	–
AcO-8	19.2, CH_3_; 169.3, C	–	–	–
AcO-15	–	19.8, CH_3_; 170.7, C	–	–
1'	128.9, C	129.1, C	130.9, C	–
2'/6'	129.6, CH	129.8, CH	129.8, CH	–
3'/5'	128.3, CH	128.1, CH	128.5, CH	–
4'	133.5, CH	133.3, CH	133.14, CH	–
1''	–	126.4, C	130.1, C	–
2''/6''	–	150.5, CH (2''); 138.0, CH (6'')	129.7, CH	–
3''/5''	–	124.0, CH (5'')	128.3, CH	–
4''	–	153.0, CH	133.11, CH	–
CO_2_-6	–	164.4, C	–	165.6, C
CO_2_-8	–	–	164.8, C	–
CO_2_-9	165.4, C	165.1, C	165.2, C	165.7, C

* Data are exchangeable.

**Figure 2:**
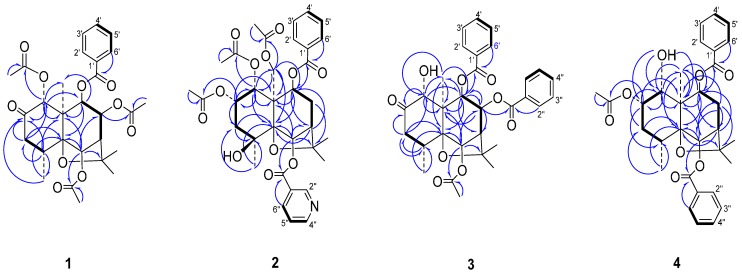
Main ^1^H-^13^C long-range correlation (⇀) and ^1^H-^1^H correlation (**—**) signals in the HMBC (Heteronuclear Multiple Bond Correlation) and COSY (^1^H-^1^H Correlation Spectroscopy) spectra of **1**–**4**.

The location sites of the ester groups and ketone carbonyl group were determined by an HMBC experiment (Figure 2). The correlations between δ_H_ 5.82 (s, H-1)/δ_C_ 169.5 (CH_3_CO_2_–), δ_H_ 5.35 (s, H-6)/δ_C_ 169.8 (CH_3_CO_2_–), δ_H_ 5.53 (dd, *J* = 3.2, 6.4 Hz, H-8)/δ_C_ 169.3 (CH_3_CO_2_–), and δ_H_ 5.16 (d, *J* = 6.4 Hz, H-9)/δ_C_ 165.4 (PhCO_2_–) showed that three acetoxy groups and one benzoyloxy group were at C-1, C-6, C-8 and C-9, respectively. The correlation between δ_H_ 5.82 (s, H-1) and δ_C_ 204.6 (C=O) as well as the downfield chemical shift and singlet of H-1 showed that position 2 was a ketone carbonyl group.

**Figure 3:**
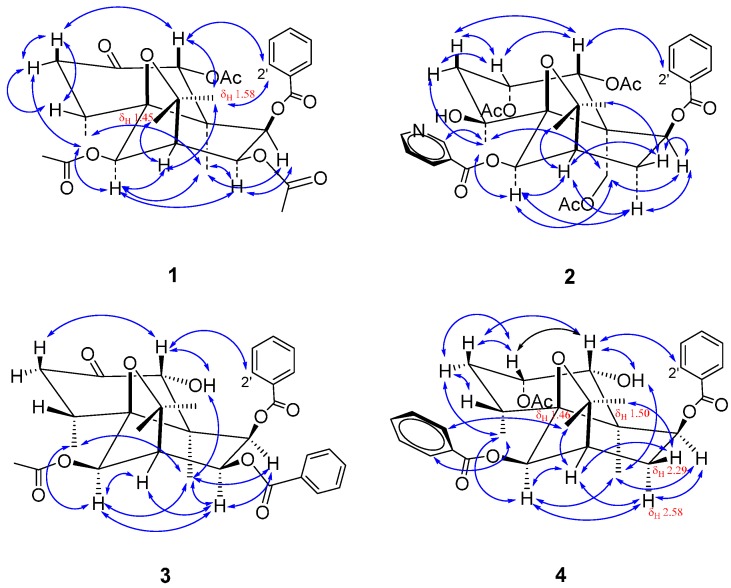
Main NOE (Nuclear Overhauser Effect) correlation signals (↔) in the NOESY (Nuclear Overhauser Enhancement Spectroscopy) spectra of **1**–**4**.

The stereochemistry of compound **1** was determined by a NOESY (Nuclear Overhauser Enhancement Spectroscopy) spectrum (Figure S5). In the NOESY spectrum (Figure 3), the correlations of δ_H_ 5.82 (s, H-1) to 3.15 (dd, *J* = 7.3, 12.9 Hz, H-3_ax_), 1.58 (s, H-13) to 7.92 (d, *J* = 8.4 Hz, H-2'), δ_H_ 5.35 (s, H-6) to 0.90 (d, *J* = 7.6 Hz, H-14), 5.53 (dd, *J* = 3.2, 6.4 Hz, H-8) to 1.22 (s, H-15), and δ_H_ 5.16 (d, *J* = 6.4 Hz, H-9) to 5.53 (dd, *J* = 3.2, 6.4 Hz, H-8) and 1.22 (s, H-15) showed that three acetoxy groups at C-1, C-6 and C-8 were equatorial and the 9-benzoyloxy group was axial. It is worth noting that 8-OH or 8-AcO in the previously isolated compounds from the same plant are axial [1], whereas the 8-AcO of compound **1** is located at an equatorial bond.

In dihydro-β-agarofuran sesquiterpene polyesters isolated from the same plant, the two six-membered rings are in *trans *configuration while both H-1 and H-6 are axial [1], which are also the general characteristics of natural dihydro-β-agarofuran sesquiterpenes [4,5]. Based on the facts above in conjunction with the above-mentioned stereochemistry analysis and biosynthesis point of view, compound **1** is identified as (1*R*,4*R*,5*S*,6*R*,7*R*,8*S*,9*R*,10*R*)-1,6,8-triacetoxy-9-benzoyloxydihydro-β-agarofuran-2-one.

Compound **2**, obtained as a pale yellow solid with [α]_D_^19^ = +18.448° (*c* = 0.23, CH_3_OH), possessed a molecular formula of C_34_H_39_NO_12_, as deduced from HR-ESI-MS data (*m*/*z*: 676.2359 [M + Na]^+^) (Figure S12) and ^13^C-NMR data. The IR showed the presence of ester carbonyl (1751, 1719 cm^−1^), hydroxyl (3551 cm^−1^) and aromatic groups (3063, 1593 cm^−1^). The UV spectrum showed the presence of benzoate moieties with maximum absorption peaks at 202, 228 and 264 nm. Spraying with potassium heptaiodobismuthate solution gave an orange-red spot on thin layer chromatographic plate, indicating **2** belonged to alkaloids.

The ^1^H- and ^13^C-NMR (Figures S7 and S8), DEPT and HSQC spectroscopic data (Figure S9) of **2** (Table 1 and Table 2) showed signals of five carbonyl groups (δ_C_ = 164.4, 165.1, 169.6, 170.0, 170.7), six methyl groups (δ_H_/δ_C_ = 1.41/24.4, 1.47/19.1, 1.49/24.7, 1.53/28.2, 1.99/19.6, 2.22/19.8, each hydrogen signal being a singlet and 3H), three methylene groups with one linked to an oxygen atom (δ_H_/δ_C_ = 1.90 (dd, *J* = 2.5, 15.0 Hz, 1H) and 2.22 (m, 1H)/41.2, 2.25 (m, 1H) and 2.59 (4 × d, *J* = 3.5, 7.0, 16.0 Hz, 1H)/34.3, 4.35 (d, *J* = 13.0 Hz, 1H) and 4.99 (d, *J* = 13.0 Hz, 1H)/65.2), five methine groups with four linked to an oxygen atom (δ_H_/δ_C_ = 6.30 (s, 1H)/79.2, 5.54 (d, *J* = 3.1 Hz, 1H)/70.9, 5.42 (d, *J* = 7.0 Hz, 1H)/68.0, 5.41 (m, 1H)/69.1, 2.33 (br s, 1H)/49.1), four quaternary sp^3^ carbon atoms with three linked to an oxygen atom (δ_C_ = 55.3, 69.7, 84.5, 90.8), two quaternary sp^2^ carbon atoms (δ_C_ 126.4, 129.1) and nine tertiary sp^2^ carbon atoms (δ_H_ = 7.39–9.23 (9H)/δ_C_ 124.0–153.0 (nine peaks)). The ^1^H-NMR, HSQC and ^1^H-^1^H COSY spectra revealed the presence of one phenyl group (δ_H_/δ_C_ = 7.39 (t, *J* = 7.7 Hz, 2H)/128.1, 7.54 (t, *J* = 7.4 Hz, 1H)/133.3, 7.94 (d, *J* = 7.8 Hz, 2H)/129.8) and one 3-pyridyl group (δ_H_ 9.23 (s, 1H)/150.5, 8.68 (br s, 1H)/153.0, 8.47 (d, *J* = 8.0 Hz, 1H)/138.0, 7.50 (dd, *J* = 2.6, 8.0 Hz, 1H)/124.0). The complete assignments of signals of all hydrogen atoms on the dihydro-β-agarofuran rings were carried out by an analysis of the ^1^H-^1^H COSY and coupling constants. The signals of H-12, H-13, H-14 and H-15 were assigned on the base of the HMBC analysis (Figure S10).

In the HMBC spectrum, the correlations between δ_H_ 5.54 (d, *J* = 3.1 Hz, 1H, H-1), 1.47 (s, 3H, 1-CH_3_C=O) and δ_C_ = 169.6, between δ_H_ = 5.41 (m, 1H, H-2), 1.99 (s, 3H, 2-CH_3_C=O) and δ_C_ = 170.0, and between δ_H_ = 4.35 (d, *J* = 13.0 Hz, 1H, H-15_a_), 4.99 (d, *J* = 13.0 Hz, 1H, H-15_b_), 2.22 (s, 3H, 15-CH_3_C=O) and δ_C_ = 170.7 revealed the presence of 1-AcO, 2-AcO and 15-AcO groups while the correlations of both δ_H_ = 6.30 (s, 1H, H-6) and 8.47 (d, *J* = 8.0 Hz, 1H, H-6'') to δ_C_ = 164.4, and both δ_H_ = 5.42 (d, *J* = 7.0 Hz, 1H, H-9) and 7.94 (d, *J* = 7.8 Hz, 2H, H-2') to δ_C_ = 165.1 indicated the presence of 6-nicotinoyloxy and 9-benzoyloxy groups. The correlations between δ_H_ = 5.41 (m, 1H, H-2), 1.90 (dd, *J* = 2.5, 15.0 Hz, 1H, H-3_eq_), 2.22 (m, 1H, H-3_ax_), 1.41 (s, 3H, H-14) and δ_C_ = 69.7 (C-4) showed that the unique hydroxyl group was located at C-4.

In the NOESY spectrum (Figure S11), the cross signals of δ_H_ = 5.54 (d, *J* = 3.1 Hz, 1H, H-1) toδ_H_ = 5.41 (m, 1H, H-2), 2.22 (m, 1H, H-3_ax_) and 7.94 (d, *J* = 7.8 Hz, 2H, H-2') showed that 1-AcO and 2-AcO groups were equatorial and axial, respectively. The correlations between δ_H_ = 1.41 (s, 3H, H-14) and 1.90 (dd, *J* = 2.5, 15.0 Hz, 1H, H-3_eq_), 4.35 (d, *J* = 13.0 Hz, 1H, H-15_a_), 4.99 (d, *J* = 13.0 Hz, 1H, H-15_b_) and 6.30 (s, 1H, H-6), and between δ_H_ = 6.30 (s, 1H, H-6) and 4.35 (d, *J* = 13.0 Hz, 1H, H-15_a_), 4.99 (d, *J* = 13.0 Hz, 1H, H-15_b_) indicated that both 4-OH and 6-nicotinoyloxy groups were located at an equatorial bond. The correlations of δ_H_ = 5.42 (d, *J* = 7.0 Hz, 1H, H-9) to 4.35 (d, *J* = 13.0 Hz, 1H, H-15_a_) and 2.59 (4 × d, *J* = 3.5, 7.0, 16.0 Hz, 1H, H-8_ax_) showed 9-benzoyloxy group was axial. Based on the NOESY analysis above as well as the fact that 1-OH or 1-ester group in all known natural dihydro-β-agarofuran sesquiterpenes were found to be equatorial, the absolute configuration of **2** was determined. Thus, **2** was elucidated as (1*R*,2*S*,4*S*,5*S*,6*R*,7*R*,9*S*,10*R*)-1,2,15-triacetoxy-9-benzoyloxy-6-nicotinoyloxy dihydro-β-agarofuran. This finding on **2** is of a certain significance because this compound is the first nitrogen-containing dihydro-β-agarofuran sesquiterpene polyester isolated from this plant.

Compound **3**, obtained as a pale yellow solid with [α]_D_^19^ = +15.364° (*c* 0.26, CH_3_OH), possessed a molecular formula of C_31_H_34_O_9_, as deduced from HR-ESI-MS data (*m*/*z*: 573.2073 [M + Na]^+^) (Figure S18) and ^13^C-NMR data (Figure S14). The IR showed the presence of carbonyl (1730 cm^−1^), hydroxyl (3466 cm^−1^), and aromatic groups (3068, 1602, 1558 cm^−1^). The UV spectrum showed the presence of benzoate moieties with maximum absorption peaks at 202, 228, 274 and 282 nm.

The ^1^H-NMR spectrum of compound **3** (Figure S13) showed the signals of one acetyl group (δ_H_ = 2.15, s, 3H), two phenyl groups with two sets of aromatic hydrogen signals (δ_H_ = 7.26–8.04, 10H) and one hydroxy group (δ_H_ = 3.19, d, *J* = 4.5 Hz) which disappeared after the addition of D_2_O. The ^13^C-NMR spectrum showed the presence of one acetic ester (δ_C_ = 21.2, 169.4) and two benzoic ester moieties (δ_C_ = 128.3–133.1, eight peaks; 164.8, 165.2). Besides these ester moieties, the ^13^C-NMR, DEPT and HSQC spectra (Figure S15) showed the presence of four methyl groups (δ_H_/δ_C_ = 1.52 (s, 3H)/31.2, 1.82 (s, 3H)/26.8, 1.01(d, *J* = 7.6 Hz, 3H)/18.4, 1.22(s, 3H)/18.9), one methylene group (δ_H_/δ_C_ = 3.24 (dd, *J* = 7.8, 13.1 Hz, 1H), 2.37 (br d, *J* = 13.1 Hz, 1H)/42.8), six methine groups with four bearing an oxygen atom (δ_H_/δ_C_ = 2.78 (d, *J* = 2.9 Hz, 1H)/53.7, 2.86 (m, 1H)/38.4, 5.19 (d, *J* = 4.5 Hz, 1H)/75.1, 5.44 (d, *J* = 6.6 Hz, 1H)/72.8, 5.49 (s, 1H)/77.3, 5.84 (dd, *J* = 3.2, 6.6 Hz, 1H)/69.9), three quaternary sp^3^ carbon atoms (δ_C_ 56.3, 83.8, 88.7) with two linked to an oxygen atom and one ketone group (δ_C_ = 211.5), which were very similar to those of the skeleton of (1*R*,4*R*,5*S*,6*R*,7*R*,8*R*,9*R*,10*R*)-1-acetoxy-6,9-dibenzoyloxy-8-hydroxydihydro-β-agarofuran-2-one isolated from the same plant [1].

Based on the cross peak (δ_H_/δ_H_ = 3.19/5.19) in the ^1^H-^1^H COSY spectrum, the same coupling constant (*J* = 4.5 Hz) in combination with the correlations of δ_H_ = 5.19 to δ_C_ = 18.9 (C-15), 56.3 (C-10), 72.8 (C-9) and 211.5 (C-2) in the HMBC spectrum, the signals at δ_H_ = 5.19 and 3.19 were assigned as H-1 and 1-OH, respectively. In the HMBC spectrum (Figure S16), the correlations of δ_H_ = 5.49 (s, 1H) to δ_C_ = 56.3 (C-10), 69.9 (C-8), 83.8 (C-11) and 88.7 (C-5), δ_H_ = 5.84 (dd, *J* = 3.2, 6.6 Hz, 1H) to δ_C_ = 53.7 (C-7) and 72.8 (C-9), and δ_H_= 5.44 (d, *J* = 6.6 Hz, 1H) to δ_C_ = 18.9 (C-15), 53.7 (C-7), 56.3 (C-10), 69.9 (C-8) and 88.7 (C-5) showed that the peaks at δ_H_ = 5.49, 5.84, 5.44 belonged to H-6, H-8 and H-9, respectively. The above assignments were further supported by the ^1^H-^1^H COSY spectrum and coupling constants. The complete assignments of the other protons and all the protonated carbons were finished on the base of the analysis of HSQC, COSY spectra and coupling constants.

The substitution sites of three ester groups were established by the analysis of the HMBC spectrum (Figure 2). Correlations between δ_H_ = 5.49 (H-6), 2.15 (C*H*_3_C=O-6) and δ_C_ = 169.4 (C=O), between δ_H_ = 5.84 (H-8), 7.78 (d, *J* = 7.5, 2H, H-2'') and δ_C_ = 164.8 (C=O), and between δ_H_ = 5.44 (H-9), 8.04 (d, *J* = 7.5 Hz, 2H, H-2') and δ_C_ = 165.2 (C=O) indicated the presence of 6-acetoxy and 8,9-dibenzoyloxy groups. The long-distance correlations between δ_H_ = 5.19 (H-1), 3.24 (H-3_ax_) and δ_C_ = 211.5 (C-2) in conjunction with downfield shift of H-3 compared with those of **2** showed that the ketone carbonyl group was located at C-2. Thus, compound **3** was identified as 6-acetoxy-8,9-dibenzoyloxy-1-hydroxydihydro-β-agarofuran-2-one.

In the NOESY spectrum (Figure S17), the correlations between δ_H_ = 5.19 (H-1) and 3.24 (H-3_ax_), 8.04 (H-2'), between δ_H_ = 3.19 (1-OH) and 1.27 (H-15), between δ_H_ = 5.49 (H-6) and 1.01 (H-14), 1.27 (H-15), 5.84 (H-8), and between δ_H_ = 5.44 (H-9) and 1.22 (H-15), 5.84 (H-8) showed that 1-OH, 6-OAc and 8-benzoyloxyl were equatorial while 9-benzoyloxyl was axial. Accordingly, compound **3** was elucidated as (1*R*,4*R*,5*S*,6*R*,7*R*,8*S*,9*R*,10*S*)-6-acetoxy-8,9-dibenzoyloxy-1-hydroxydihydro-β-agarofuran-2-one. Interestingly, compound **3** is one diastereomer of the known compound triptogelin A-4 ((1*R*,4*R*,5*S*,6*R*,7*R*,8*R*,9*S*,10*S*)-6-acetoxy-8,9-dibenzoyloxy-1-hydroxydihydro-β-agarofuran-2-one) (([α]_D_^23^ = –31.0° (*c* 1.0, CH_3_OH)), isolated from the plant *Tripterygium wilfordii *Hook fil. var. *regelii *Makino [6]. The only difference between two compounds lies in the configurations of C-8 and C-9, which is supported by their specific rotations.

Compound **4**, obtained as a white solid with [*α*]_D_^21^ = +3.152° (*c* 0.24, CHCl_3_), possessed a molecular formula of C_31_H_36_O_8_, as deduced from ESI-MS data (*m/z*: 559.23 [M+Na]^+^) (Figure S24) and ^13^C-NMR data. The IR showed the presence of ester carbonyl (1718 cm^−1^), hydroxyl (3519 cm^−1^) and aromatic groups (3062, 3010, 1602, 1584 cm^−1^). The UV spectrum showed the presence of benzoate moieties with maximum absorption peaks at 242, 274 and 282 nm.

The ^1^H- and ^13^C-NMR spectrum data (Figures S19 and S20) of compound **4** are identical to those of compound wightianine A ((1*S*,2*R*,4*R*,5*S*,6*R*,7*R*,9*S*,10*S*)-2-acetoxy-6,9-dibenzoyloxy-1-hydroxydihydro-β-agarofuran) isolated from the same plant [3], which suggests that compound **4** should be 2-acetoxy-6,9-dibenzoyloxy-1-hydroxydihydro-β-agarofuran. This inference was further confirmed by the HSQC and HMBC spectra (Figures S21 and S22). However, the NOESY spectrum (Figure S23) showed that compound **4** should have different stereochemistry from wightianine A. In the NOESY spectrum, the strong correlations between δ_H_ = 4.62 (dd, *J* = 4.0, 5.4 Hz, 1H, H-1) and 5.34 (q-like, *J* = 3.4 Hz, 1H, H-2), 2.41 (4 × d, *J* = 3.4, 6.2, 13.0 Hz, 1H, H-3_ax_), 8.09 (d, *J* = 8.4 Hz, 2H, H-2' and H-6') and between δ_H_ = 1.82 (d, *J *= 5.5 Hz, 1H, 1-OH) and 1.42 (s, 3H, H-15) showed that 1-OH and 2-AcO groups were equatorial and axial, respectively. The correlations between δ_H_ = 5.67 (s, 1H, H-6) and 1.21 (d, *J *= 7.7 Hz, 3H, H-14), 1.42 (s, 3H, H-15) and between δ_H_ = 5.08 (d, *J* = 7.0 Hz, 1H, H-9) and 1.42 (s, 3H, H-15), 2.58 (4 × d, *J* = 3.2, 7.0, 16.4 Hz, 1H, H-8_ax_) showed that the 6-benzoyloxy group was equatorial while 9-benzoyloxy group was axial. Accordingly, compound **4** was identified as (1*R*,2*S*,4*R*,5*S*,6*R*,7*R*,9*S*,10*S*)-2-acetoxy-6,9-dibenzoyloxy-1-hydroxydihydro-β-agarofuran. The only difference of **4** with wightianine A [3] is the epimerization at C-1 and C-2. The C-1 configuration in compound **4** is in agreement with the stereochemical characteristics of this class of compounds in which H-1 generally has the axial configuration [7].

In addition to four new compounds (**1**–**4**), six known dihydro-β-agarofuran derivatives (**5**–**10**) were also isolated and characterized by comparison of their physical and spectral data with those in the literature [1] as (1*R*,4*R*,5*S*,6*R*,7*R*,9*S*,10*R*)-1-acetoxy-6,9-dibenzoyloxydihydro-β-agarofuran-2-one (**5**), (1*R*,2*S*,4*R*,5*S*,6*R*,7*R*,8*R*,9*R*,10*S*)-1,2,6,8,15-pentaacetoxy-9-benzoyloxydihydro-β-agarofuran (**6**), (1*R*,2*S*,4*R*,5*S*,6*R*,7*R*,9*S*,10*R*)-1,2,6,15-tetraacetoxy-9-benzoyloxydihydro-β-agarofuran (**7**), (1*R*,4*R*,5*S*,6*R*,7*R*,8*R*,9*R*,10*R*)-1-acetoxy-6,9-dibenzoyloxy-8-hydroxydihydro-β-agarofuran-2-one (**8**), (1*R*,2*S*,4*R*,5*S*,6*R*,7*R*,8*R*,9*R*,10*R*)-1,2-diacetoxy-6,9-dibenzoyloxy-8-hydroxydihydro-β-agarofuran (**9**) and (1*R*,2*S*, 4*R*,5*S*,6*R*,7*R*,9*S*,10*R*)-1-acetoxy-6,9-dibenzoyloxy-2-hydroxydihydro-β-agarofuran (**10**).

New compounds **1**–**4** were screened to explore their influence on the proliferation of MKN-45 cells, NB4 cells, and MCF-7 cells by MTT assay. *Cis*-platinum (*cis*-diaminodichloroplatinum, DDP) as a standard anticancer drug purchased from Sigma Aldrich Co. (St. Louis, MO, USA) was used as a positive control drug. Based on the cytotoxic activities of compounds **5**, **7**–**10** [1], the tested concentrations of the compounds were set as 20 μM. The cytotoxicity data were shown in Table 3.

**Table 3:** Inhibition percentages of compounds **1**–**4** against three cancer cell lines at 20 μM (48 h).

Compound	NB4	MKN-45	MCF-7
**1**	12.7 ± 3.2	8.9 ± 2.3	6.0 ± 0.9
**2**	13.7 ± 5.4	9.3 ± 1.6	2.9 ± 1.4
**3**	27.1 ± 3.3	9.9 ± 1.9	8.3 ± 1.4
**4**	85.6 ± 3.4	30.5 ± 4.5	4.5 ± 1.4
*cis*-diaminodichloroplatinum	100.0 ± 0.0	57.1 ± 2.2	44.2 ± 3.5

Almost all the test compounds showed some activities against three tested cell lines at the concentration of 20 μM, but were obviously weaker than DDP. Among all the test compounds, **4** showed the higher activity on NB4 with the inhibition rate of 85.6% and MKN-45 cells with the inhibition rate of 30.5% followed by compound **3**. Compounds **1** and **2** showed the similar activity against each of the cell lines at 20 μM. For susceptibility of various cell lines, all the compounds showed the order of NB4 cells > MKN-45 cells > MCF-7 cells. In our previous study on the cytotoxic activity of **5**, **7**–**10**, it was found that the presence of hydroxyl groups could improve the activity [1]. Based on the fact above, we speculated that the higher activity of **4** and **3** compared to **1** or **2** may be related to the presence of hydroxyl groups in the structures of **4** and **3**.

The sesquiterpene polyesters with a dihydroagarofuran skeleton have attracted considerable attention from synthetic organic chemists and pharmacologists due to their complex and diverse chemical structures and biological activities, such as insect antifeedant and/or insecticidal activity [8,9], cytotoxic activity [1], antitumor promoting activity [10], antitubercular [11], immunosuppressive [12], anti-HIV [13], anti-inflammatory activity [14] and reversal of the multidrug resistance (MDR) phenotype [15]. To date, hundreds of dihydro-β*-*agarofuran compounds have been isolated from dozens of species of plants. It is noteworthy that most of the natural dihydro-β*-*agarofurans mainly originated from plants of the Celastraceae family [5]. Only a minority were found in other family plants such as Lamiaceae [1]. Nevertheless, the present study and previous works on phytochemistry of *P. wightiana* [1,2,3] leading to the discovery of 12 new and 4 known dihydro-β*-*agarofuran sesquiterpene polyesters suggest that this plant may be an important resource of dihydro-β*-*agarofuran sesquiterpenes.

## 3. Experimental Section

### 3.1. General

Melting points were determined on an XT-4 micro-melting point apparatus and were uncorrected. Optical rotations were measured with an Autopol V instrument (Rudolph Research Analytical, Hachettstown, NJ, USA). Infrared (IR) spectra were recorded in wave numbers (cm^−1^) on a Bruker TENSOR 27 transform infrared spectrophotometer with KBr disks (Bruker Daltonics Inc., Bremen, Germany). ^1^H-NMR spectra at 400 or 500 MHz and ^13^C-NMR spectra at 100 or 125 MHz were recorded with a Bruker AVANCE III spectrometer with TMS (Bruker Daltonics Inc., Bremen, Germany) as internal standard. Coupling constants were reported in hertz (Hz). Electrospray ionization (ESI-MS) was measured on a Thermo LCQ Fleet instrument (Thermo Fisher Scientific Co., Waltham, MA, USA). High-resolution mass spectra (HR-MS) were determined on a MicroTOF-QII Bruker instrument (Bruker Daltonics Inc., Bremen, Germany). Analytic preparative HPLC was carried out using a Shimadzu LC-20AD instrument equipped with an Agilent ZORBAX Eclipse Plus C_18_ column (Shimadzu Corporation, Kyoto, Japan) (5 µm, 4.6 mm × 150 mm) and SPD-20A UV/vis detector, and using methanol-water (80:20) as a mobile phase. Preparative HPLC was carried out using a Shimadzu LC-8A instrument equipped with a SHIM-PACK PRC-ODS column (Shimadzu Corporation, Kyoto, Japan, 5 μM, 20 mm × 250 mm) and SPD-M10Avp photodiode array detector and using methanol-water (80:20) as a mobile phase. Column chromatographic (CC) separations and preparative thin layer chromatography (TLC) were performed using silica gel (CC, 200–300 mesh; TLC, 300–400 mesh) (Qingdao Marine Chemical Ltd., Qingdao, China) as adsorbent materials.

### 3.2. Experimental Procedures

Plant Material. The whole plants of *P. wightiana* were collected from Qinling Mountains, Shaanxi Province, China, in June 2013, and identified by Fang Miao (College of Life Science, Northwest A&F University). A voucher specimen (No. 20061202) is deposited in the botanic specimen center of Northwest A&F University, Yangling, China.

#### 3.2.1. Extraction and Isolation

Powdered and air-dried materials of *Parnassia wightiana * (800 g) were successively extracted with ethyl acetate at 50 °C three times (3 × 2 h). After removal of solvent *in vacuo*, the black viscous residue (84 g) was treated with 400 mL methanol to remove insoluble black oily deposits. The methanol-soluble fraction (57 g) was subjected to silica gel column chromatography (Ф 35 × 457 mm) eluting with PE–AcOEt (stepwise, 10:0, 5:1, 2:1, 1:1, 0:1, each 1000 mL) and finally methanol (1000 mL). Six corresponding fractions were obtained of F1 (7.55 g), F2 (7.78 g), F3 (8.50 g), F4 (7.02 g), F5 (7.00 g) and F6 (8.61 g). F1 as dark green oils was subjected to silica gel column chromatography using PE–AcOEt (stepwise, 5:1, 1500 mL; 2:1, 1:1, each 500 mL; 0:1, 200 mL) and finally methanol (200 mL) to provide six subfractions of F11 (0.529 g), F12 (0.426 g), F13 (0,914 g), F14 (1.392 g), F15 (2.312 g) and F16 (1.495 g) by TLC analysis. F14 was separated by preparative silica gel TLC using PE–AcOEt (10:1) as developing solvents with repeatedly developing three times to yield **5** (61 mg) with R*_f_* = 0.22 (PE–AcOEt = 5:1).

To F2 (7.78 g) was added 20 mL ethanol-water mixture (4:1, *v*/*v*). The resulting solution was treated with ultrasonic wave for 10 min and stood for 6 h at room temperature. Filtration provided the filtrate and the deposit. The filtrate was used to preparative HPLC using MeOH–H_2_O (80:20 over 40 min, 10 mL·min^−1^) to yield eight subfractions of F21 (43 mg, t*_R_* = 8.45–10.10 min), F22 (212 mg, t*_R_* = 10.90–11.90 min), F23 (124 mg, t*_R_* = 12.32–12.56 min), F24 (1197 mg, t*_R_* = 12.77–15.50 min), F25 (82 mg, t*_R_* = 18.10–20.50 min), F26 (33 mg, t*_R_* = 21.75–22.30 min), F27 (348 mg, t*_R_* = 22.70–23.48 min), F28 (131 mg, t*_R_* = 24.05–26.30 min). The deposit was re-dissolved in 50 mL methanol, concentrated to *ca.* (circa) 5 mL under reduced pressure and stood overnight. Compound **6** (148 mg) was precipitated from the solution, and washed with methanol several times to afford pure **6** (148 mg) with R*_f_* = 0.34 (PE–acetone 5:1). The mother solution after removal of **6** was subjected to preparative silica gel TLC using PE–Acetone (5:1) as developing solvents to afford another pure **6** (63 mg) and **7** (64 mg) with R*_f_* = 0.45 (PE–acetone 5:1).

F21 (43 mg) was separated by preparative silica gel TLC using toluene–acetone (20:1) with repeatedly developing three times to obtain pure **1** (11 mg). The similar preparative silica gel TLC was used to separate F23 (124 mg) using toluene-acetone (10:1) to yield pure **7** (31 mg) with R*_f_* = 0.53 (toluene–acetone 10:1) and crude **8** (54 mg). The latter was again subjected to preparative silica gel TLC using toluene–acetone (20:1) to obtain pure **8** (21 mg) with R*_f_* 0.41 (toluene–acetone 10:1).

F24 (1197 mg) was isolated by silica gel column chromatography (Ф 20 × L 305 mm) using toluene–acetone (stepwise, 100:1, 1000 mL; 50:1, 500 mL; 5:1, 500 mL) to yield crude **7** and the mixture of **7** and **3**, respectively. Preparative silica gel TLC using toluene–acetone (10:1) with repeatedly developing four times was used to isolate the mixture of **7** and **3** (444 mg) to obtain pure **3** (5.1 mg) with R*_f_* = 0.44 (toluene–acetone 10:1) and crude **7**. Combined crude **7** was further subjected to preparative silica gel TLC using the same developing solvents to afford pure **7** (167 mg) with R*_f_* = 0.51 (toluene–acetone 10:1).

F28 (131 mg) was separated by preparative silica gel TLC using toluene-acetone (10:1) with repeatedly developing 2 times to obtain pure **5** (8 mg) with R*_f_* = 0.66 (toluene–acetone 10:1) and crude **4** (63 mg). The latter was again subjected to preparative silica gel TLC using PE–AcOEt (20:1) to provide pure **4** (29 mg) with R*_f_* = 0.42 (toluene–acetone 10:1).

F3 (8.50 g) dissolved in 20 mL of methanol–water (4:1) was separated by preparative HPLC using a MeOH–H_2_O (80:20 over 40 min, 10 mL·min^−1^) to yield eight subfractions of F31 (59 mg, t*_R_* = 5.72–7.58 min), F32 (104 mg, t*_R_* = 8.30–9.42 min), F33 (359 mg, t*_R_* = 9.68–10.60 min), F34 (720 mg, t*_R_* = 10.87–11.91 min), F35 (1304 mg, t*_R_* = 12.49–14.34 min), F36 (120 mg, t*_R_* = 15.30–17.04 min), F37 (30 mg, t*_R_* = 19.90–22.23 min) and F38 (103 mg, t*_R_* = 23.40–26.26 min). The same treatments as descripted for F3 were used for F5 (7.00 g) to yield subfractions of F51 (653 mg, t*_R_* = 5.20–5.60 min), F52 (153 mg, t*_R_* = 5.85–6.73 min), F53 (77 mg, t*_R_* = 7.15–7.53 min), F54 (30 mg, t*_R_* = 8.27–8.58 min), F55 (82 mg, t*_R_* = 9.19–9.78 min), F16 (38 mg, t*_R_* = 10.26–10.75 min), F57 (55 mg, t*_R_* = 11.61–12.13 min) and F58 (75 mg, t*_R_* = 12.86–14.35 min). F35 was chromatographed in a silica gel column (Ф 20 × L 305 mm) (Ф 20 × L 305 mm) using toluene–acetone (stepwise, 40:1, 800 mL; 10:1, 200 mL) and finally methanol (100 mL) to yield crude **8** (856 mg) which was further purified by preparative silica gel TLC using toluene–acetone (10:1) to obtain pure **8** (694 mg) with R*_f_* = 0.72 (PE–AcOEt 2:1).

F36 (120 mg) was subjected to preparative silica gel TLC separation using toluene-acetone (5:1) with repeatedly developing three times to provide crude **9** which was further purified by preparative silica gel TLC using toluene-acetone (10:1) with successively developing two times to obtain pure **9** (25 mg) with R*_f_* = 0.40 (toluene–acetone 10:1). The same steps as described for F36 were used to separate F38 (103 mg), and yielded pure **10** (48 mg) with R*_f_* = 0.41 (toluene–acetone 10:1). F57 (55 mg) was purified by preparative silica gel TLC using toluene–acetone (5:1) with successively developing three times to obtain pure **2** (15 mg) with R*_f_* = 0.40 (toluene–acetone 5:1).

(−)-(1*R*,4*R*,5*S*,6*R*,7*R*,8*S*,9*R*,10*R*)-1,6,8-Triacetoxy-9-benzoyloxydihydro-β-agarofuran-2-one (**1**)

Compound **1**: pale yellow solids; mp 70.0–71.2 °C; [α]_D_^19^ = −8.661° (*c* 0.26, CH_3_OH); UV (MeOH): λ_max_ (log ε) 204 (3.85), 232 (3.90), 274 (2.89), 282 (2.81) nm; IR *ν*_max_ (KBr) 3063, 2963, 2927, 1754, 1734, 1602, 1454, 1396, 1371, 1262, 1230, 1105, 1029, 973, 801, 712 cm^−1^; ^13^C-NMR and ^1^H-NMR data, see Table 1 and Table 2. (+)-HR-ESI-MS *m*/*z* 553.2035 [M + Na]^+^ (calculated for C_28_H_34_NaO_10_, 553.2050).

(+)-(1*R*,2*S*,4*S*,5*S*,6*R*,7*R*,9*S*,10*R*)-1,2,15-Triacetoxy-9-benzoyloxy-6-nicotinoyloxydihydro-β-agarofuran (**2**)

Compound **2**: pale yellow solids; mp 85.6–87.2 °C; [α]_D_^19^ = +18.448° (*c* 0.23, CH_3_OH); UV (MeOH): λ_max_ (log ε) 202 (4.14), 228 (4.12), 264 (3.43) nm; IR *ν*_max_ (KBr) 3551, 3063, 2962, 2929, 1751, 1719, 1593, 1368, 1279, 1096, 1025, 802, 713 cm^−1^; ^13^C-NMR and ^1^H-NMR data, see Table 1 and Table 2. (+)-HR-ESI-MS *m*/*z* 676.2359 [M + Na]^+^ (calculated for C_34_H_39_NNaO_12_, 676.2370).

(+)-(1*R*,4*R*,5*S*,6*R*,7*R*,8*S*,9*R*,10*S*)-6-Acetoxy-8,9-dibenzoyloxy-1-hydroxydihydro-β-agarofuran-2-one (**3**)

Compound **3**: pale yellow solids; mp 99.6–101.1 °C; [α]_D_^19^ = +15.364° (*c* 0.26, CH_3_OH); UV (MeOH): λ_max_ (log ε) 202 (4.16), 228 (4.13), 274 (3.10), 282 (3.00) nm; IR *ν*_max_ (KBr) 3466, 3068, 2963, 2924, 2854, 1730, 1602, 1558, 1283, 1263, 1233, 1105, 1028, 802, 710 cm^−1^; ^13^C-NMR and ^1^H-NMR data, see Table 1 and Table 2. (+)-HR-ESI-MS *m*/*z* 573.2073 [M + Na]^+^ (calculated for C_31_H_34_NaO_9_, 573.2101).

(+)-(1*R*,2*S*,4*R*,5*S*,6*R*,7*R*,9*S*,10*S*)-2-Acetoxy-6,9-dibenzoyloxy-1-hydroxydihydro-β-agarofuran (**4**)

Compound **4**: white solids; mp 70.8–72.1 °C; [α]_D_^19^ = +3.152° (*c* 0.24, CHCl_3_); UV (MeOH): λ_max_ (log ε) 242 (3.99), 274 (3.16), 282 (3.09) nm; IR *ν*_max_ (KBr) 3519, 3062, 3010, 2963, 2929, 1718, 1602, 1584, 1452, 1389, 1365, 1276, 1106, 1024, 892, 860, 803, 712 cm^−1^; ^13^C-NMR and ^1^H-NMR data, see Table 1 and Table 2. (+)-ESI-MS *m*/*z* 559.23 [M + Na]^+^.

#### 3.2.2. *In Vitro* Cytotoxicity Assay

Cytotoxic activity of new compounds **1**–**4** was determined according to our reported MTT (3-(4,5-dimethylthiazol-2-yl)-2,5-diphenyltetrazolium bromide) method [16]. The human gastric cell line MKN-45, human acute promyelocytic leukemia cell line NB4 and breast cancer cell line MCF-7 purchased from The Fourth Military Medical University (Xi’an, China) were used as tested cells. Briefly, MKN-45 cells and NB4 cells were cultured in DMEM (high glucose) and RPMI 1640 medium, respectively, supplemented with 10% fetal bovine serum, penicillin (100 U/mL) and streptomycin (100 μg/mL) at 37 °C in 5% CO_2_. The tested compounds were dissolved in dimethyl sulfoxide (DMSO) and diluted freshly before each experiment to 20 μM concentration in DMEM (high glucose) medium for MKN-45 or MCF-7 cells or RPMI 1640 medium for NB4 cells. The final concentration of DMSO in all experiments was 0.1% (*v*/*v*) and had negligible effect on the measured parameters.

MKN-45 or MCF-7 cells at exponential growth phase were seeded into a 96-well plate at 4.8 × 10^3^ cells/200 μL/well and cultured for 24 h. After removal of the medium, the cells in each well were treated with 200 μL solution containing 20 μM of the tested compounds and vehicle controls which only received an equivalent amount of DMSO in quintuplicate for at 37 °C 48 h. For NB4 cells, the cell suspension at exponential growth phase was treated with the solution of the tested compounds, and seeded into a 96-well plate at 4.2 × 10^4^ cells/200 μL/well in quintuplicate and cultured for 48 h. Thirty microliters of MTT solution (5 mg/mL in PBS) was added to each well with 150 μL fresh medium for an additional 4 h. After removing the supernatant, 150 μL of DMSO was added to completely dissolve the formazan crystals that had formed in viable cells in the wells. Finally, the plates were shaken and the absorbance (A) was determined using microplate reader (Bio-Rad 680) at 570 nm. The wells containing the same media as other test wells but no cells were used as blank controls. All the doses were tested in quintuplicate and the experiments were repeated at least three times. The inhibition rates (IRs) were calculated according to the following formula and expressed as means ± S.D.

IRs% = (Acontrol − Aexperimental)/(Acontrol − Ablank) × 100(1)

## 4. Conclusions

In this study we isolated four new and six known dihydro-β-agarofuran sesquiterpene polyesters from *Parnassia wightiana* and elucidated the structures of the new compounds through spectroscopic analysis including UV, IR, ^1^H-NMR, ^13^C-NMR, 2D-NMR and HR-MS. The first dihydro-β-agarofuran sesquiterpene alkaloid (**2**) was found in this plant.

## Supplementary Materials

Supplementary materials can be found at http://www.mdpi.com/1422-0067/16/05/9119/s1.
