# Supplementary material for: Further Study on Chemical Constituents of Parnassia wightiana Wall: Four New Dihydro-β-agarofuran Sesquiterpene Polyesters
Source: Int J Mol Sci. 2015 Apr 23;16(5):9119–33. doi: 10.3390/ijms16059119 (PMC4463581; doi:10.3390/ijms16059119)
Supplement: Supplementary file 1 [file ijms-16-09119-s001.pdf]

Supplementary Information

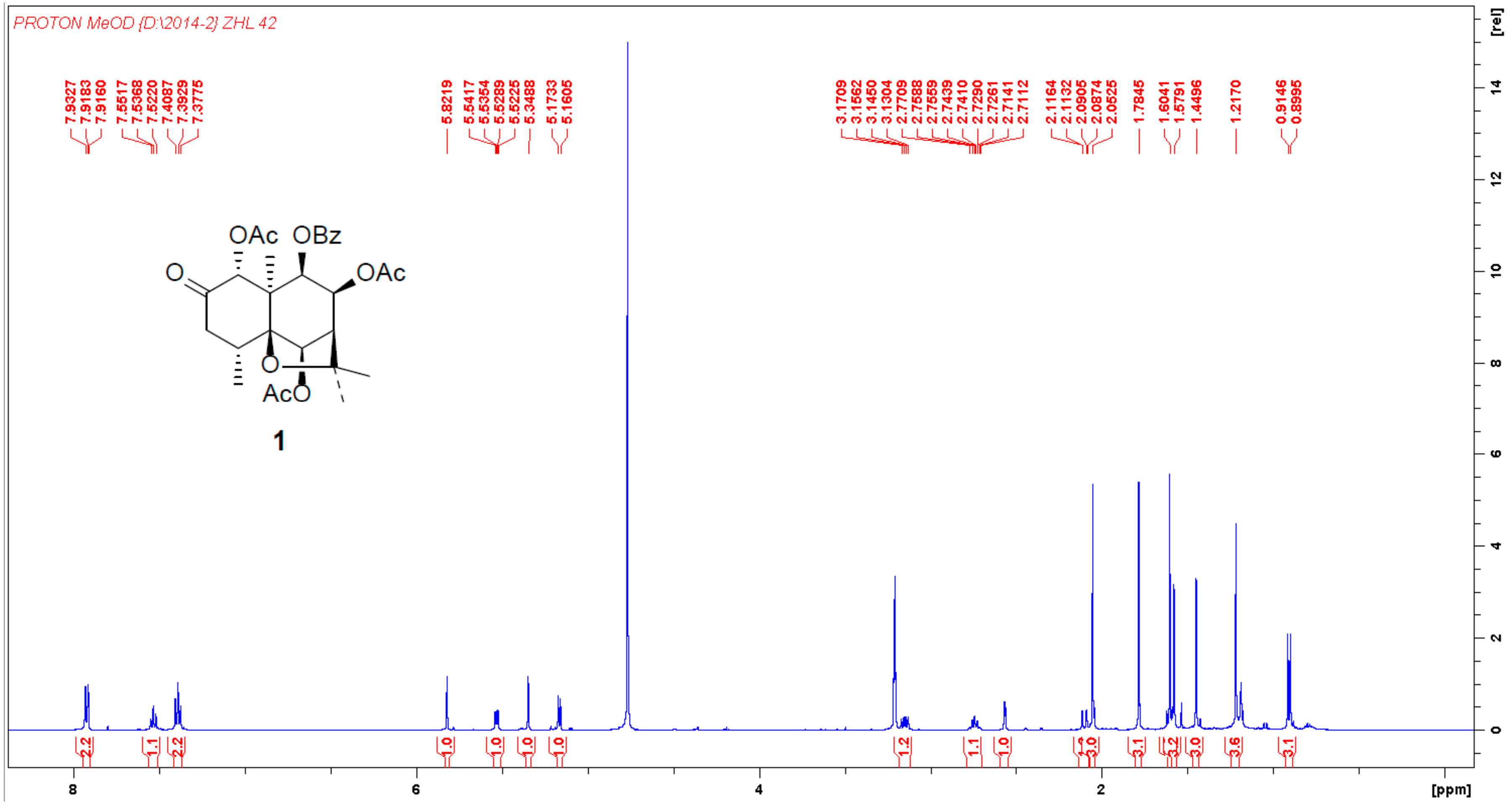

Figure S1. ^{1}H-NMR (CD3OD, 500 MHz) spectrum of **1**.

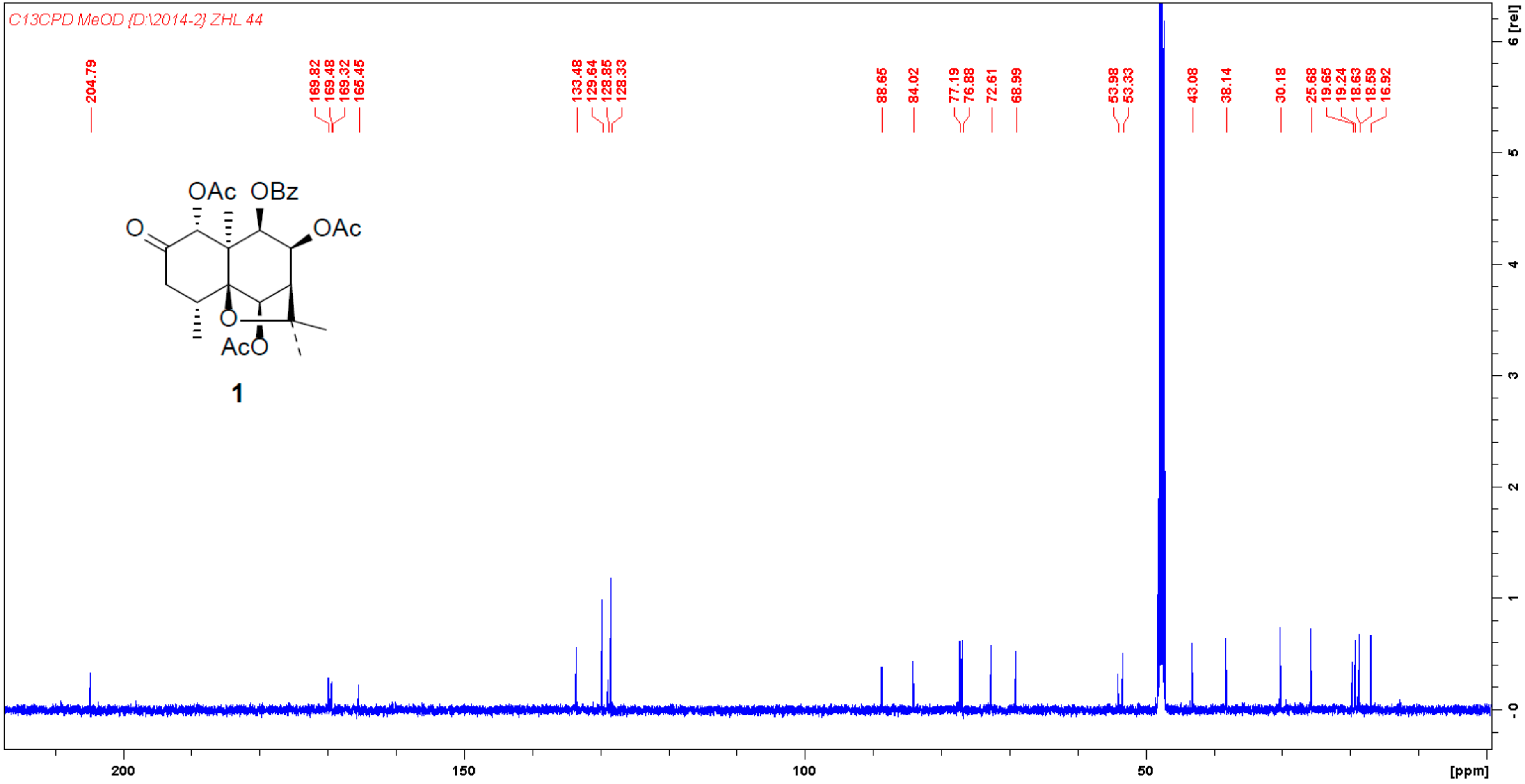

Figure S2. ^{13}C-NMR (CD_3OD, 125 MHz) spectrum of **1**.

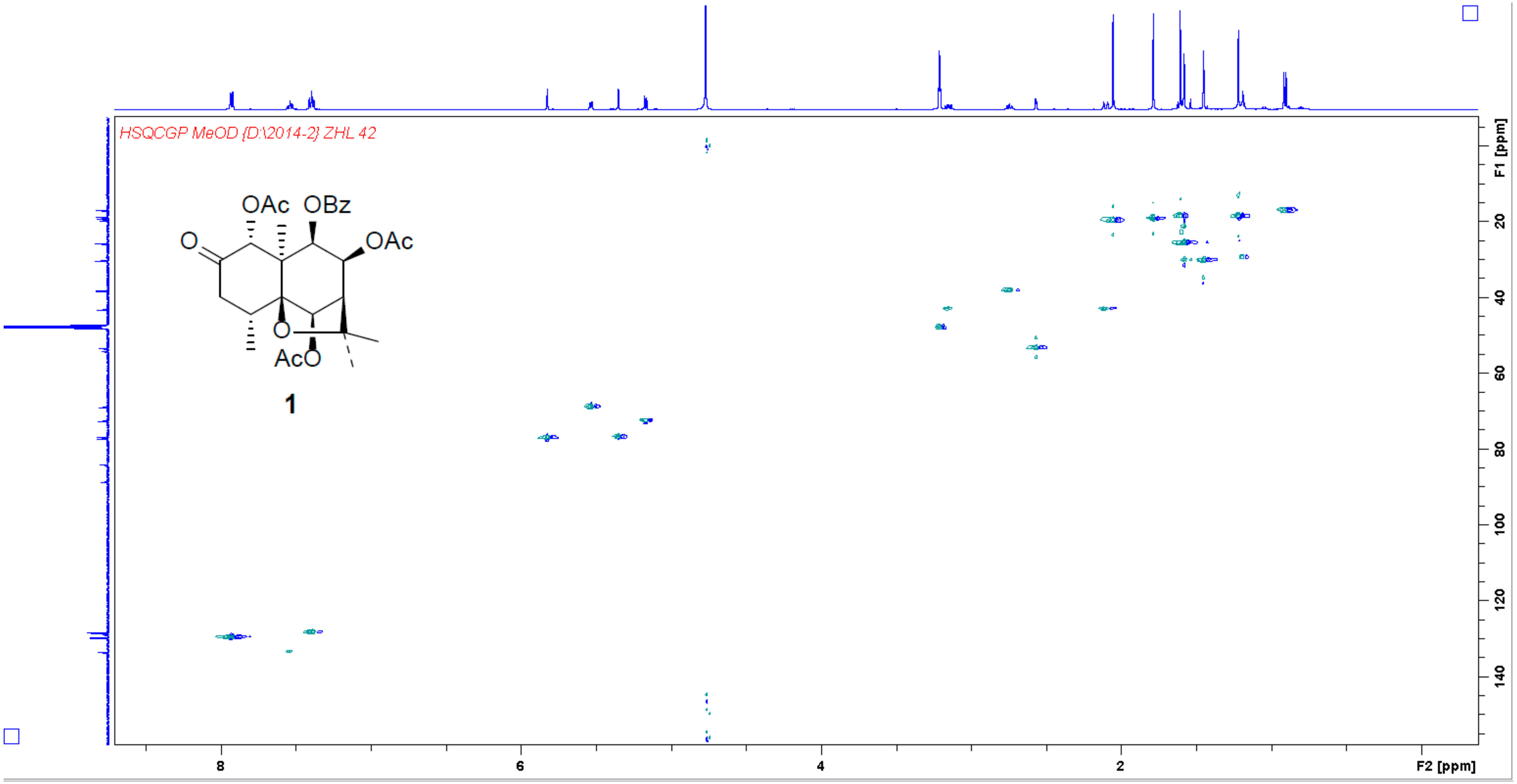

Figure S3. HSQC spectrum of **1**.

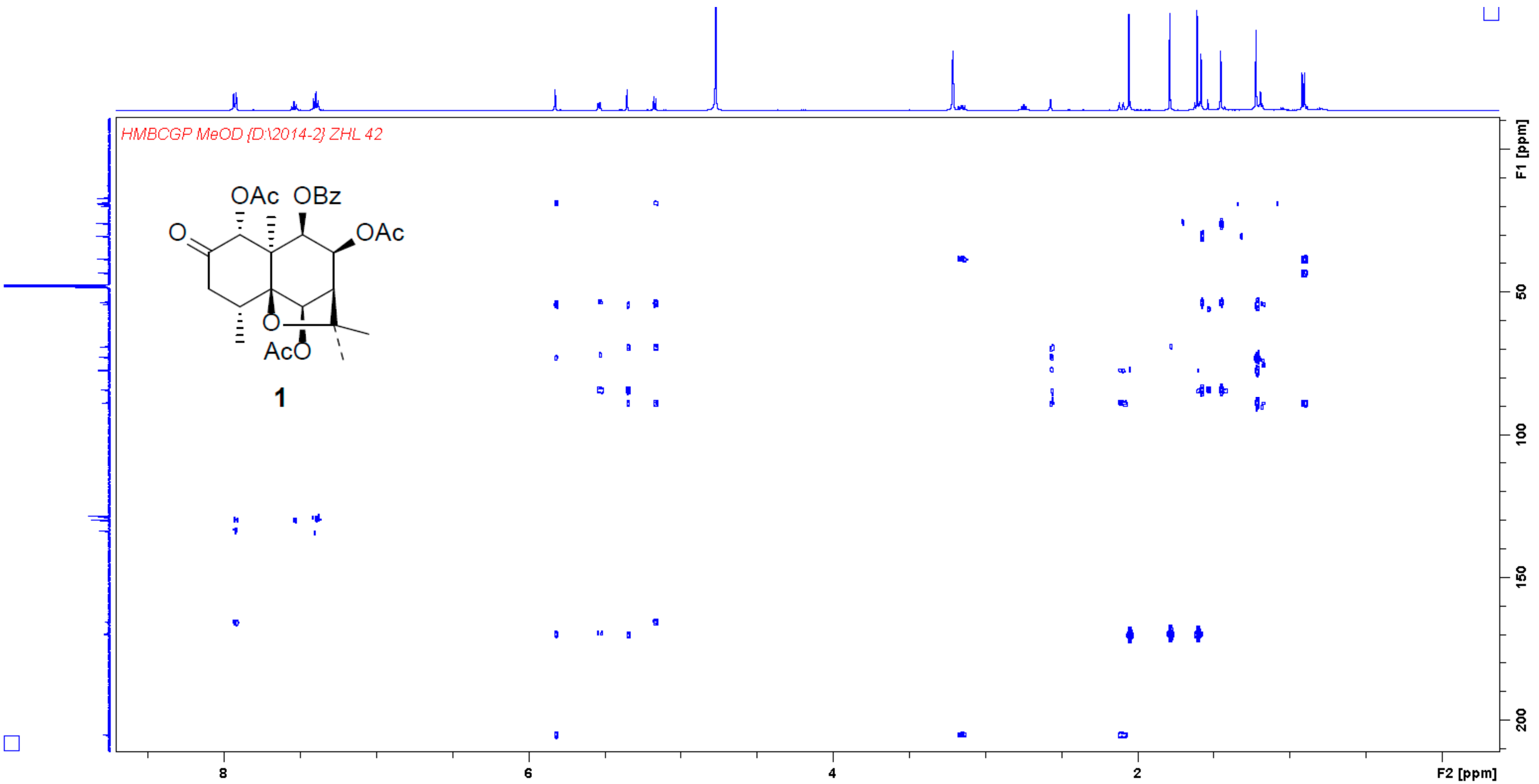

Figure S4. HMBC spectrum of **1**.

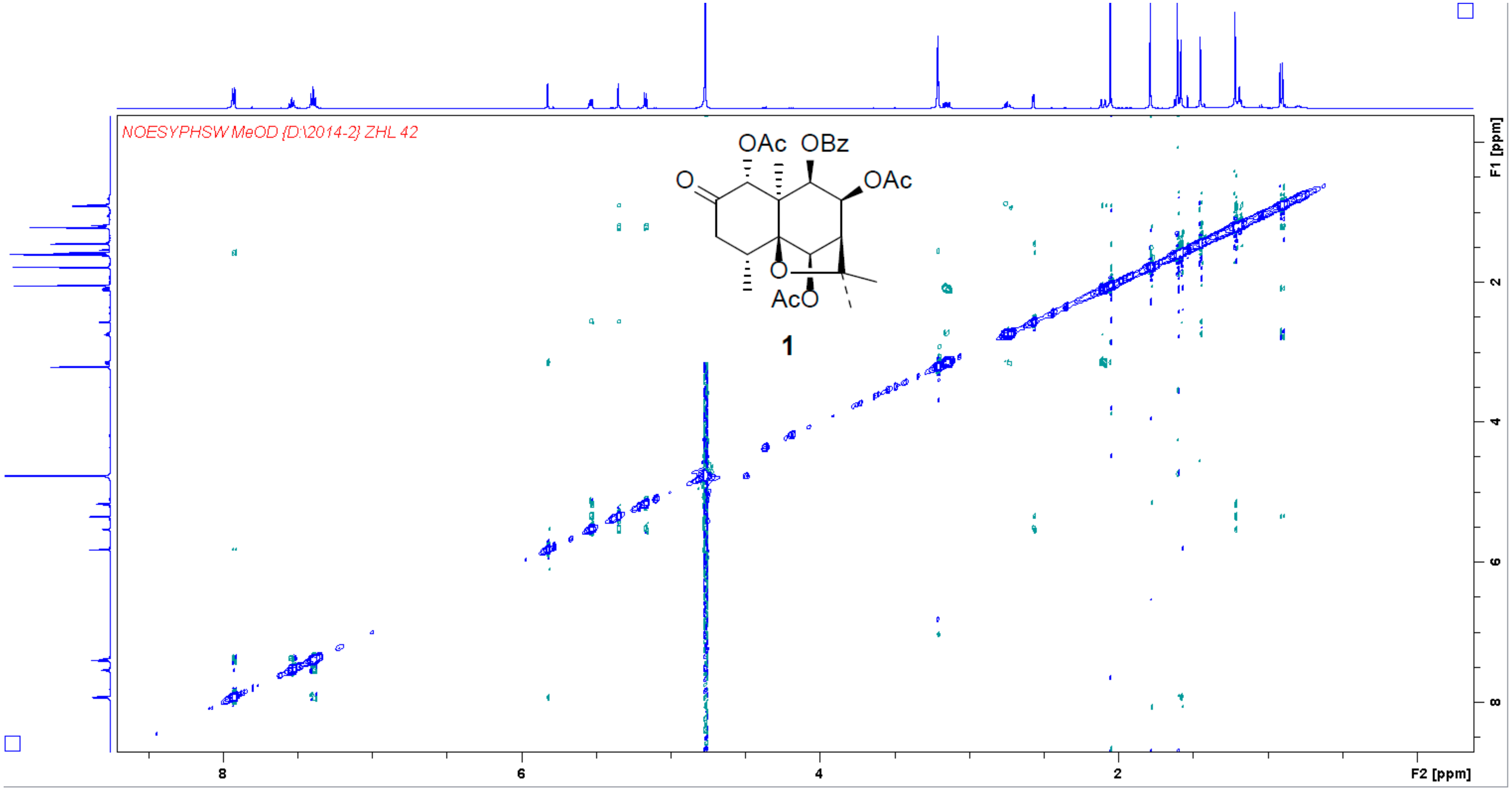

Figure S5. NOESY spectrum of **1**.

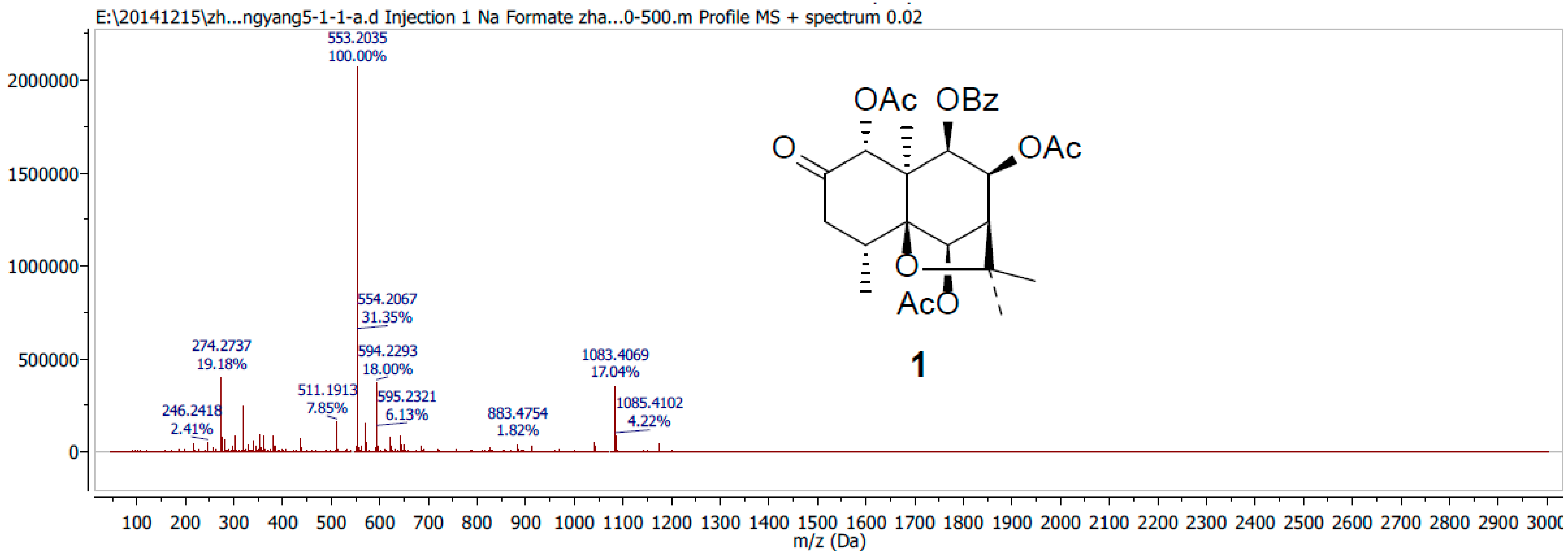

Figure S6. High-resolution mass spectrum of **1**.

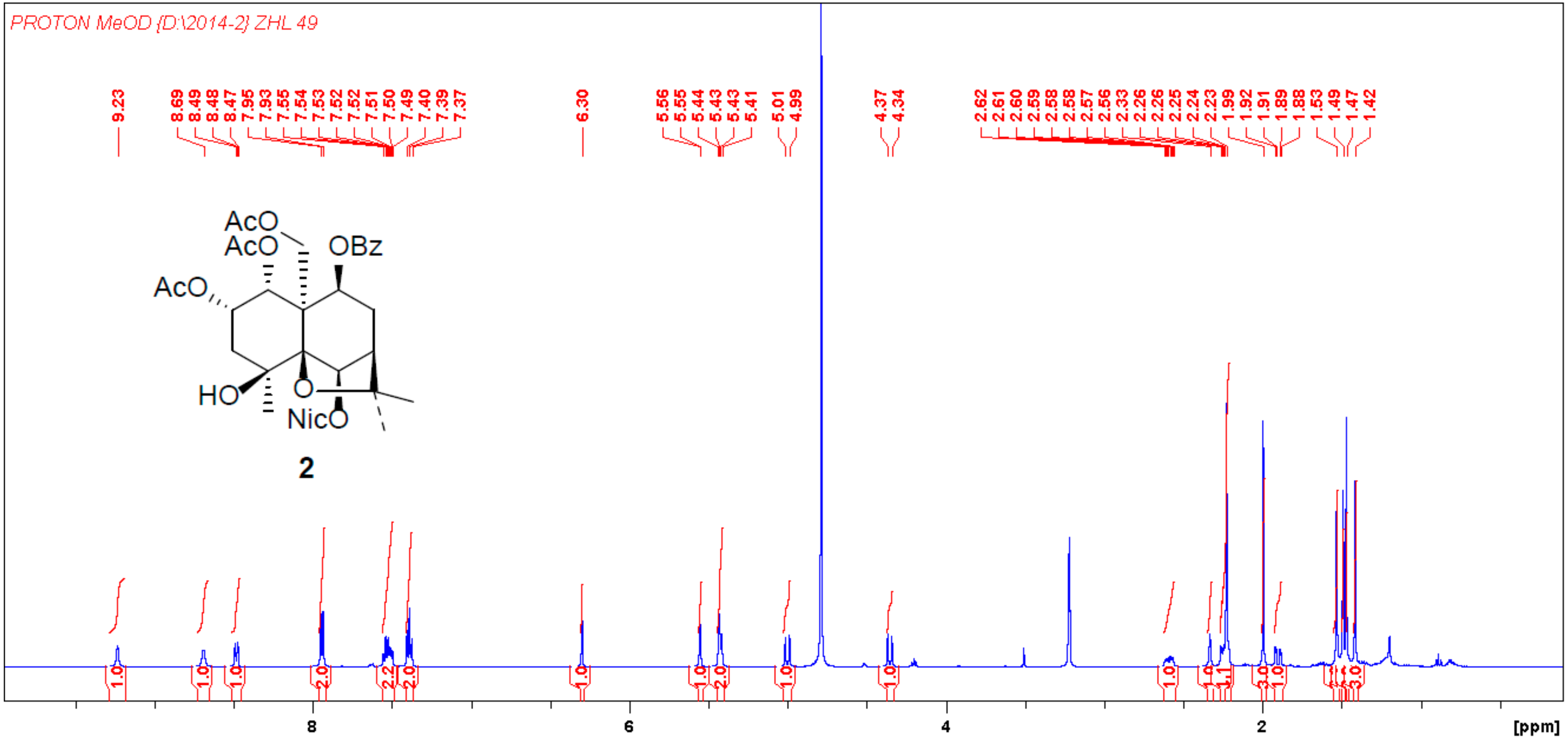

Figure S7. ^{1}H-NMR (CD_3OD, 500 MHz) spectrum of **2**.

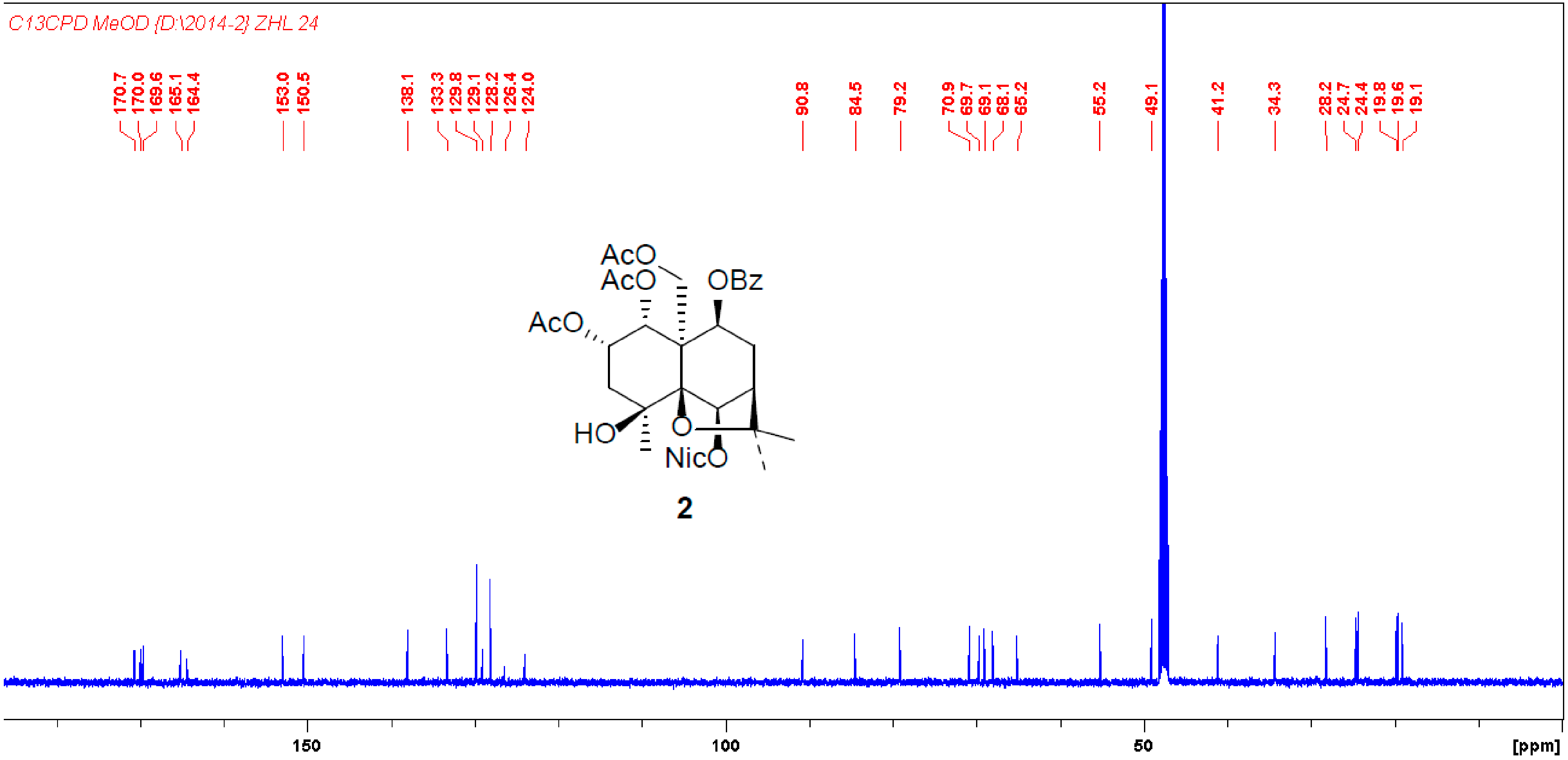

2

Figure S8. ^{13}C-NMR (CD_3OD, 125 MHz) spectrum of **2**.

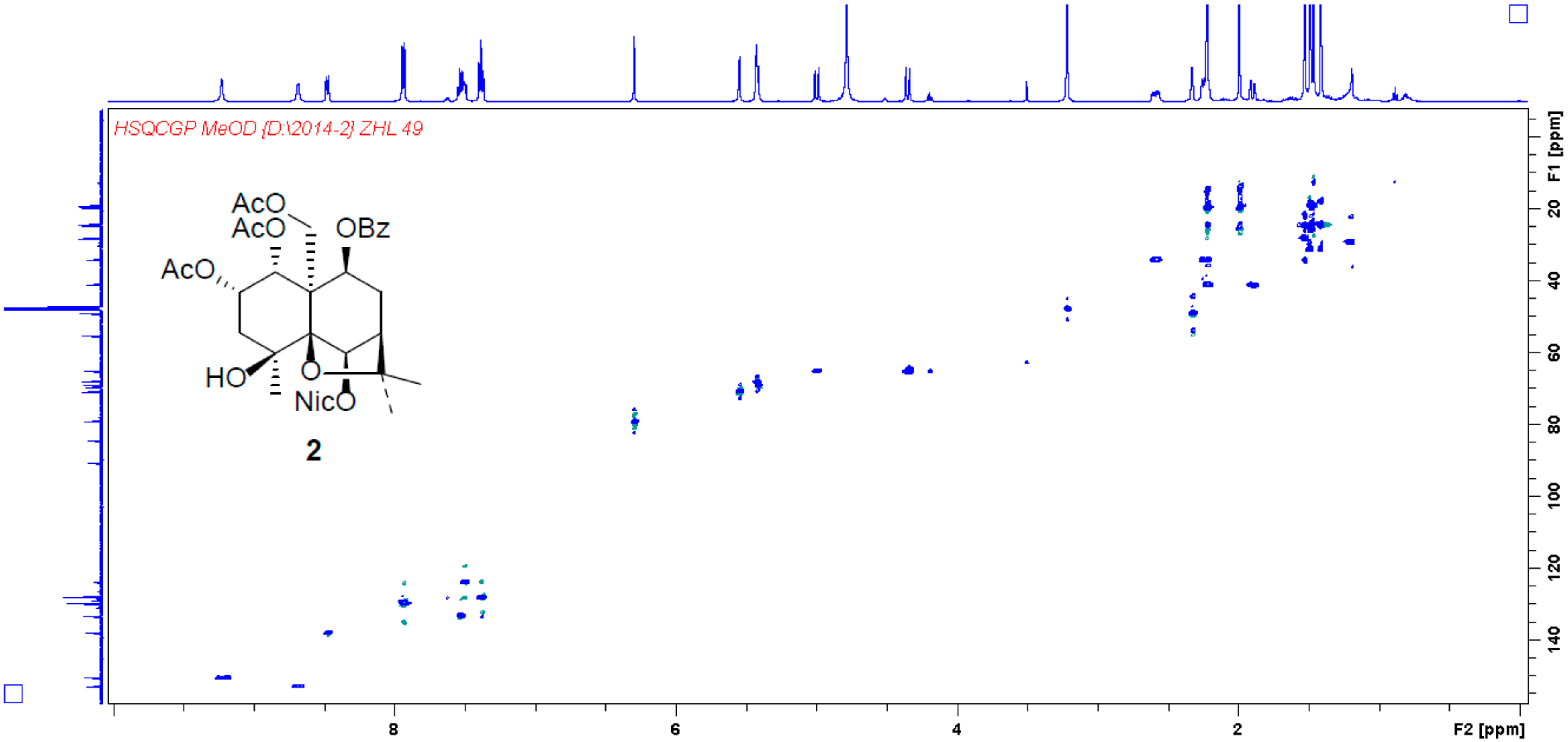

Figure S9. HSQC spectrum of **2**.

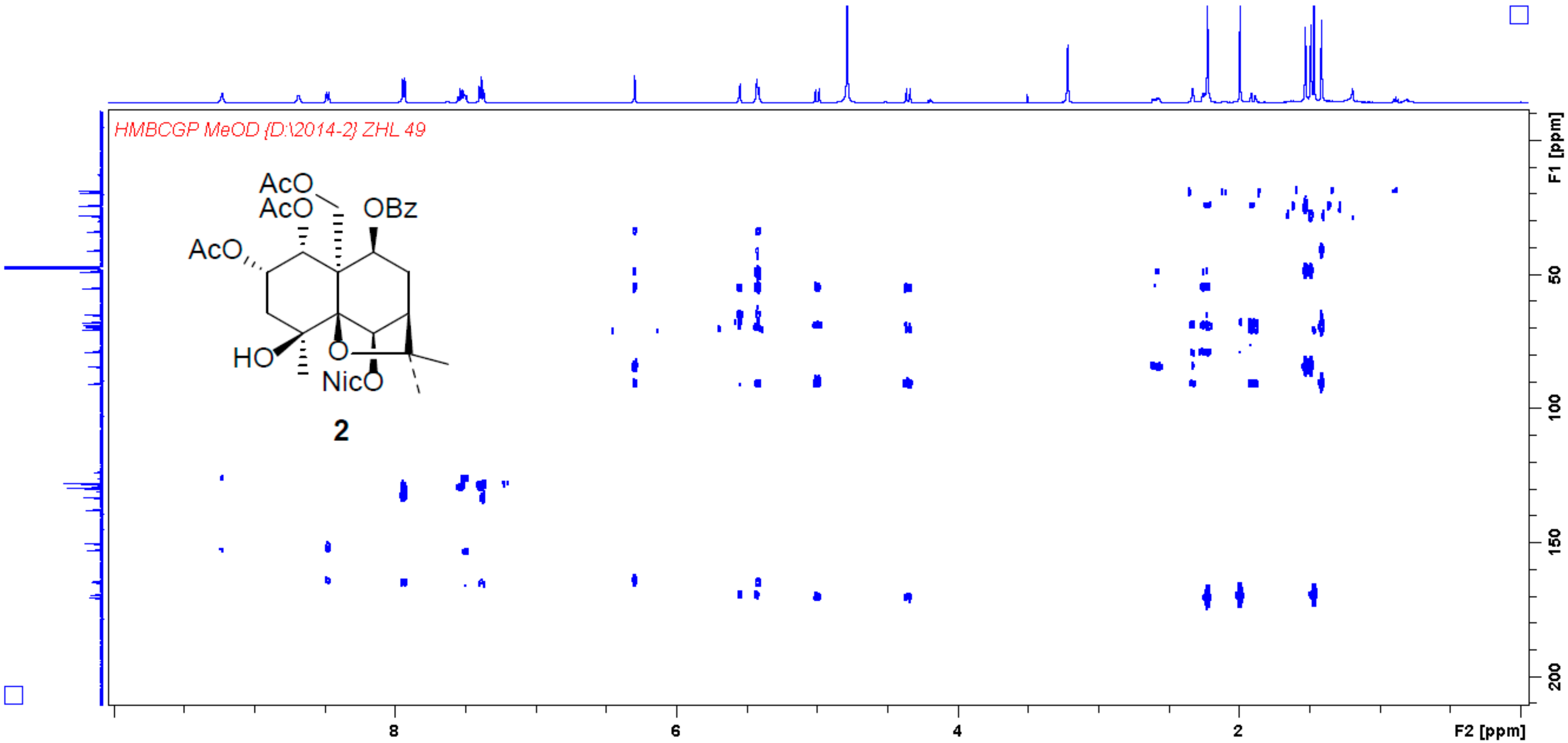

Figure S10. HMBC spectrum of **2**.

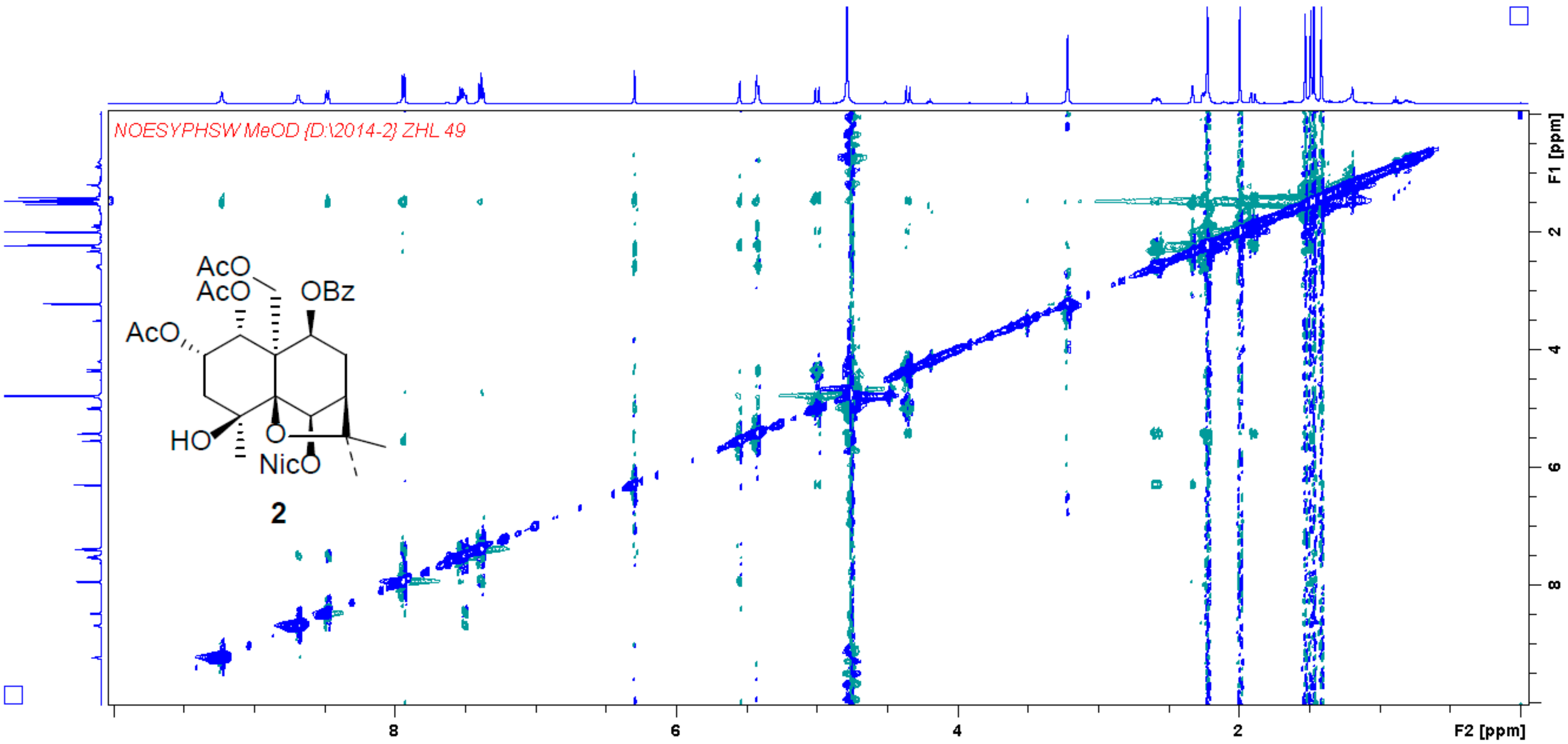

Figure S11. NOESY spectrum of **2**.

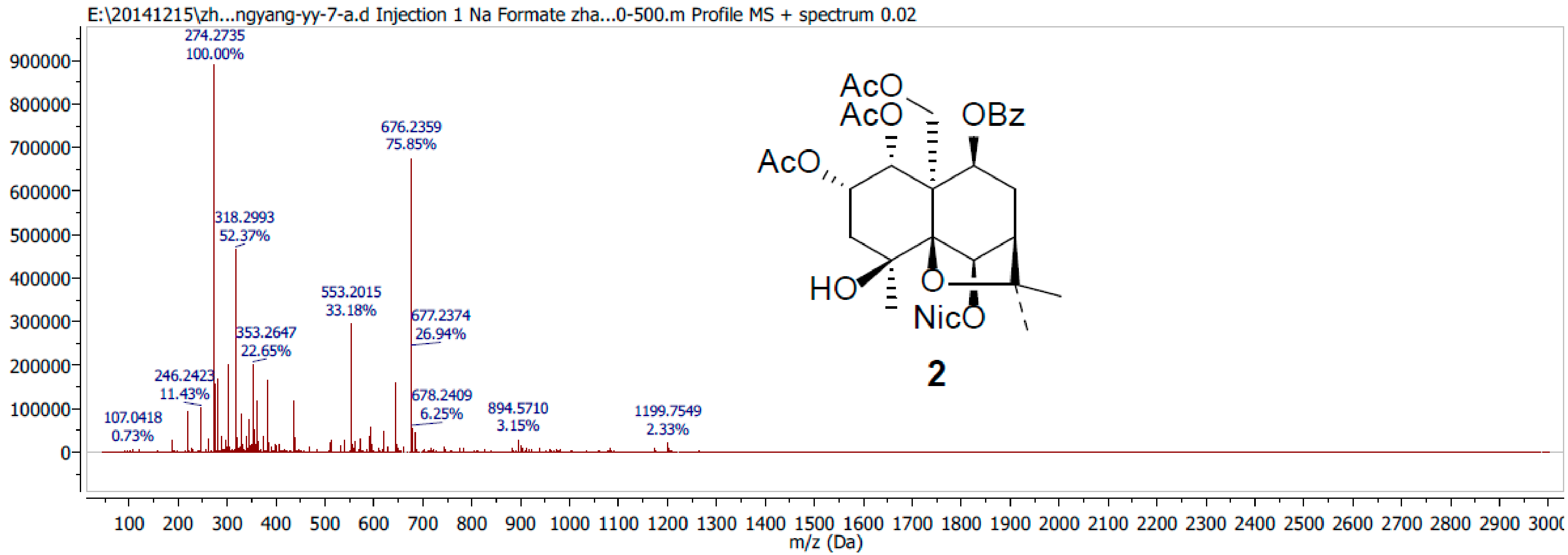

Figure S12. High-resolution mass spectrum of **2**.

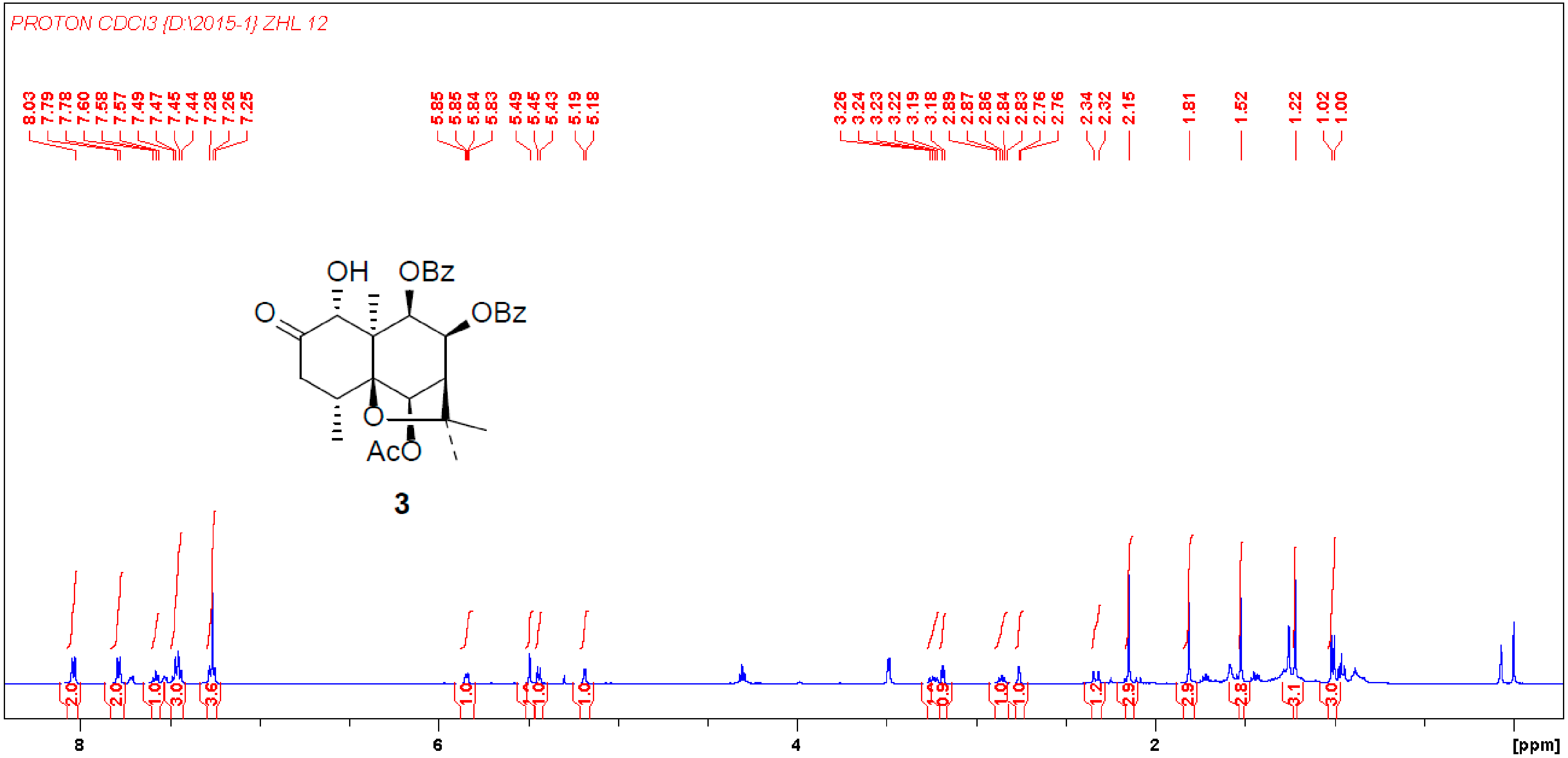

Figure S13. ^{1}H-NMR ($CDCl_3$, 500 MHz) spectrum of **3**.

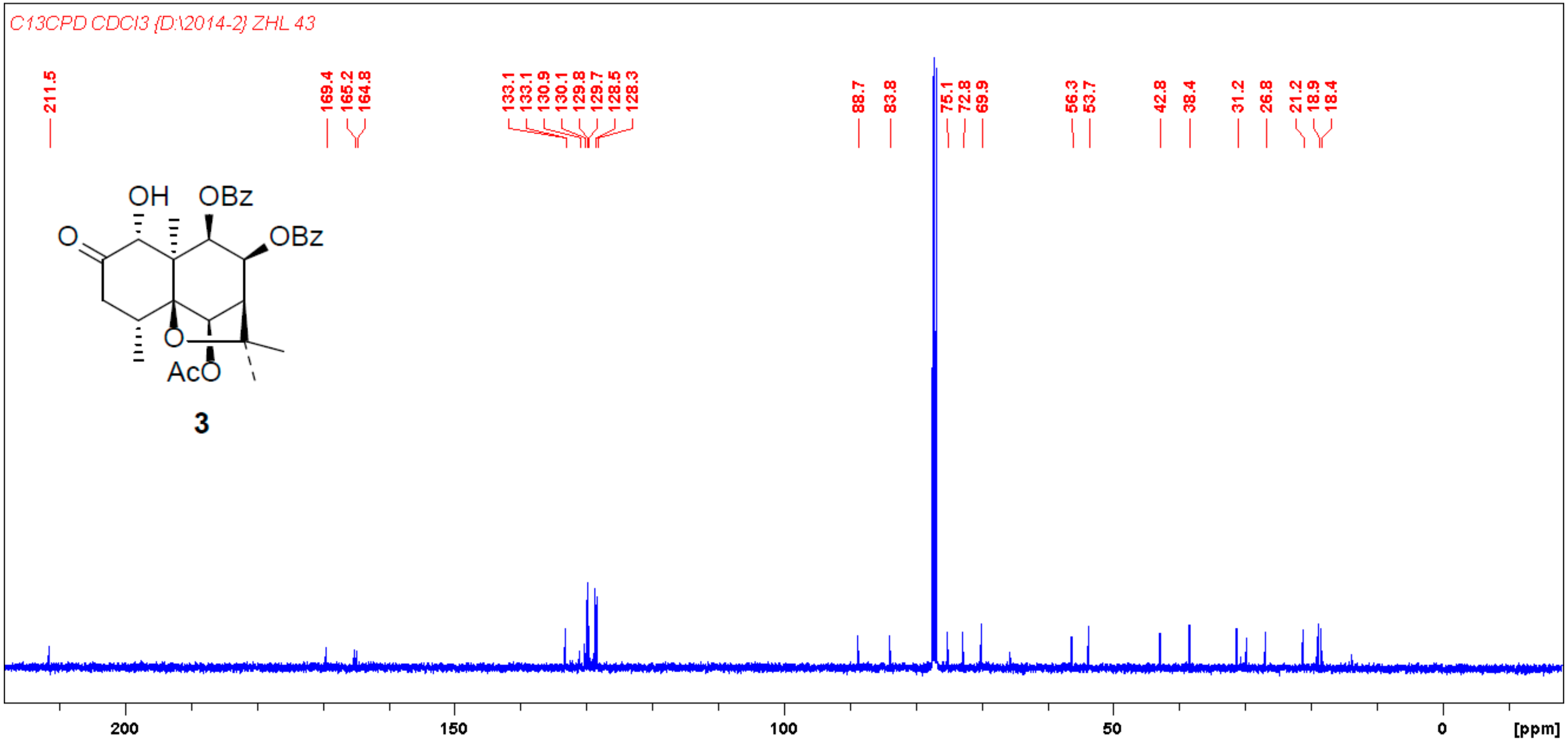

Figure S14. ^{13}C-NMR ($CDCl_3$, 125 MHz) spectrum of **3**.

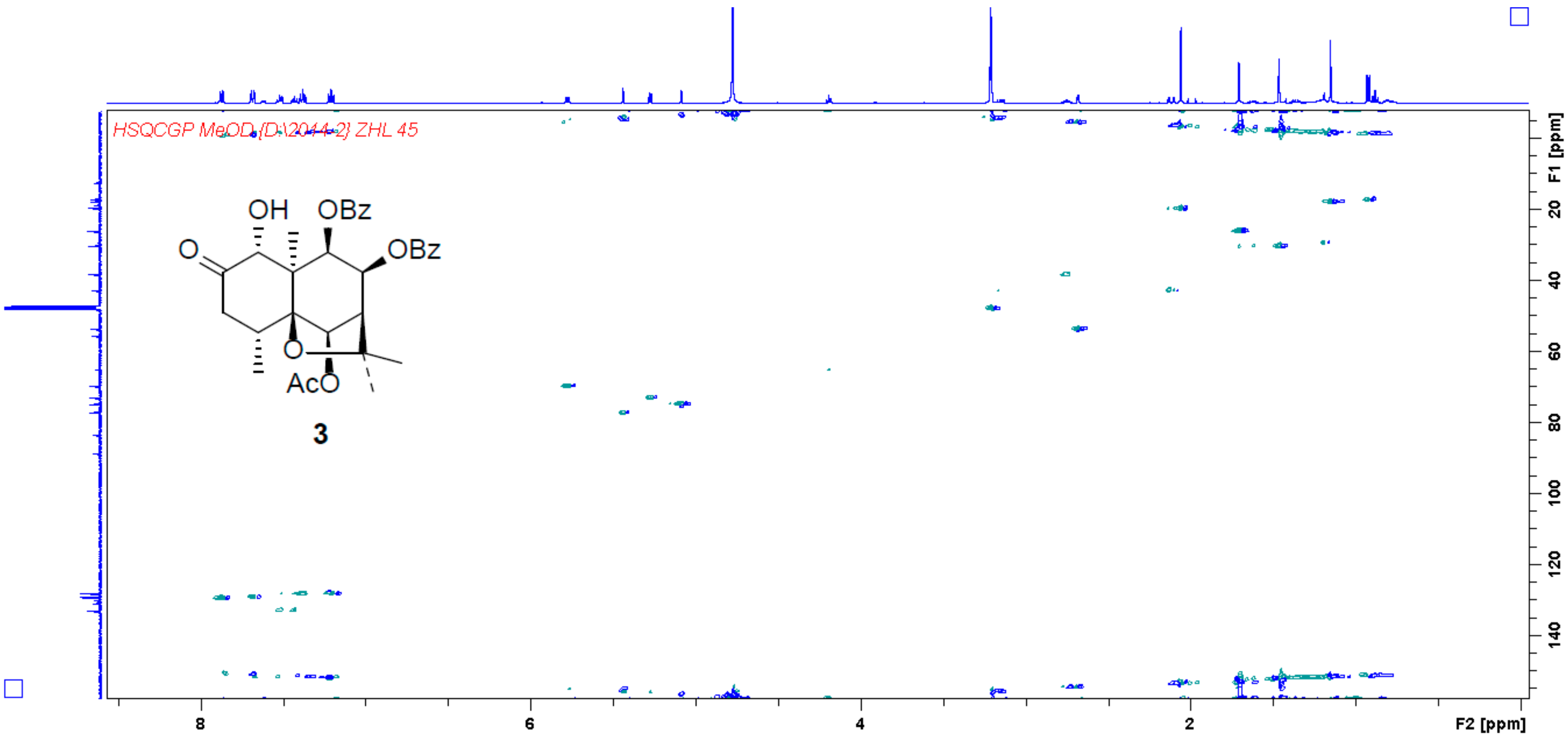

Figure S15. HSQC spectrum of **3**.

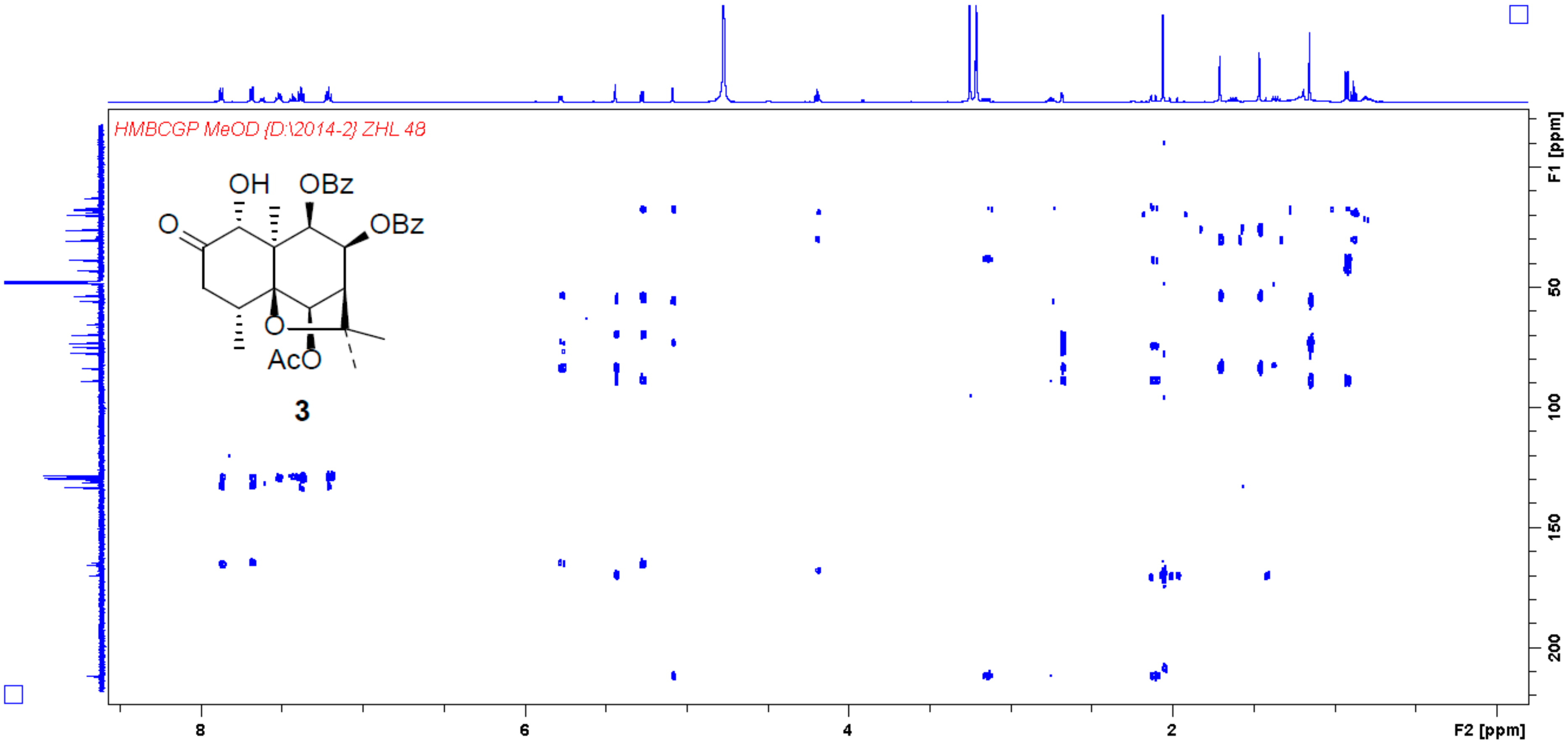

Figure S16. HMBC spectrum of **3**.

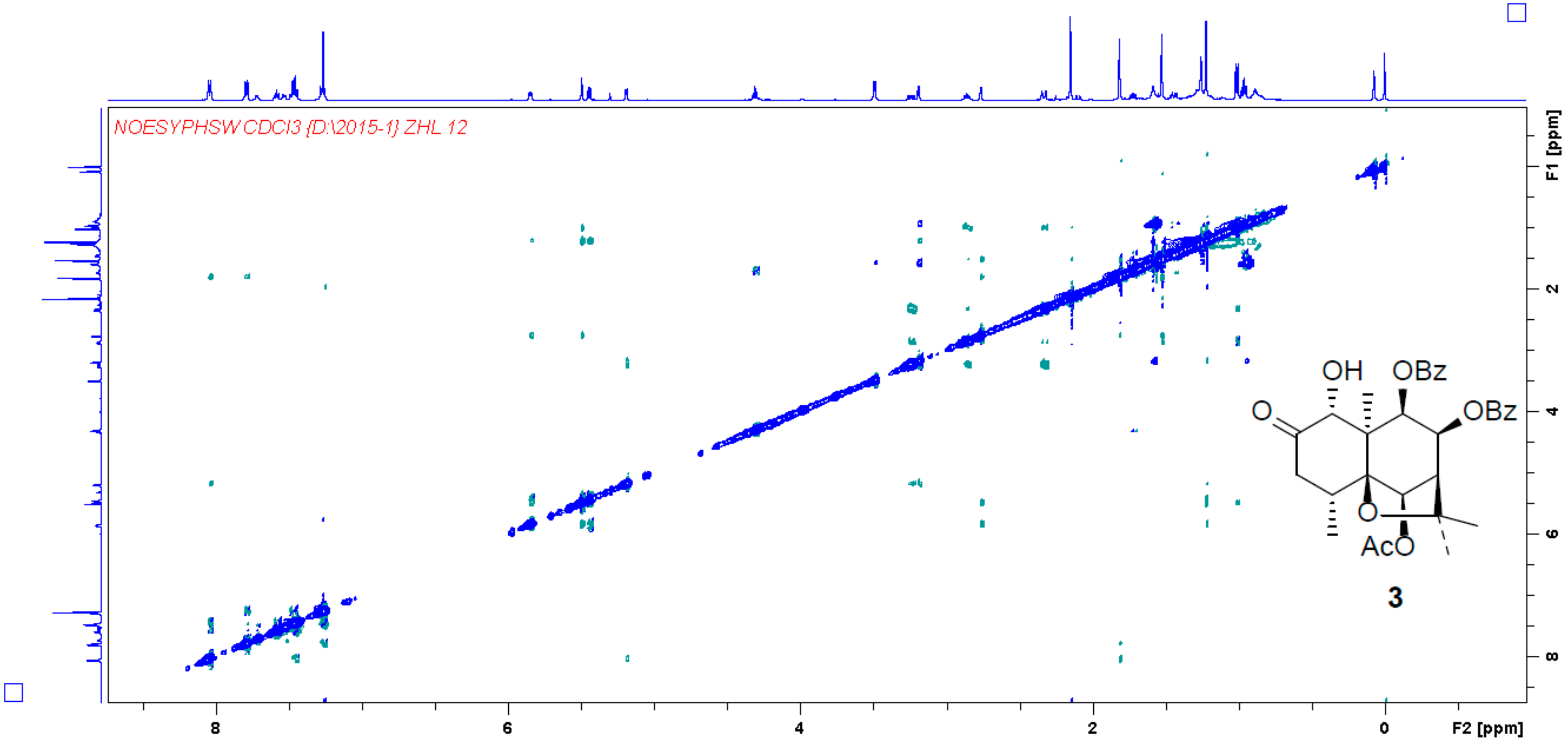

Figure S17. NOESY spectrum of **3**.

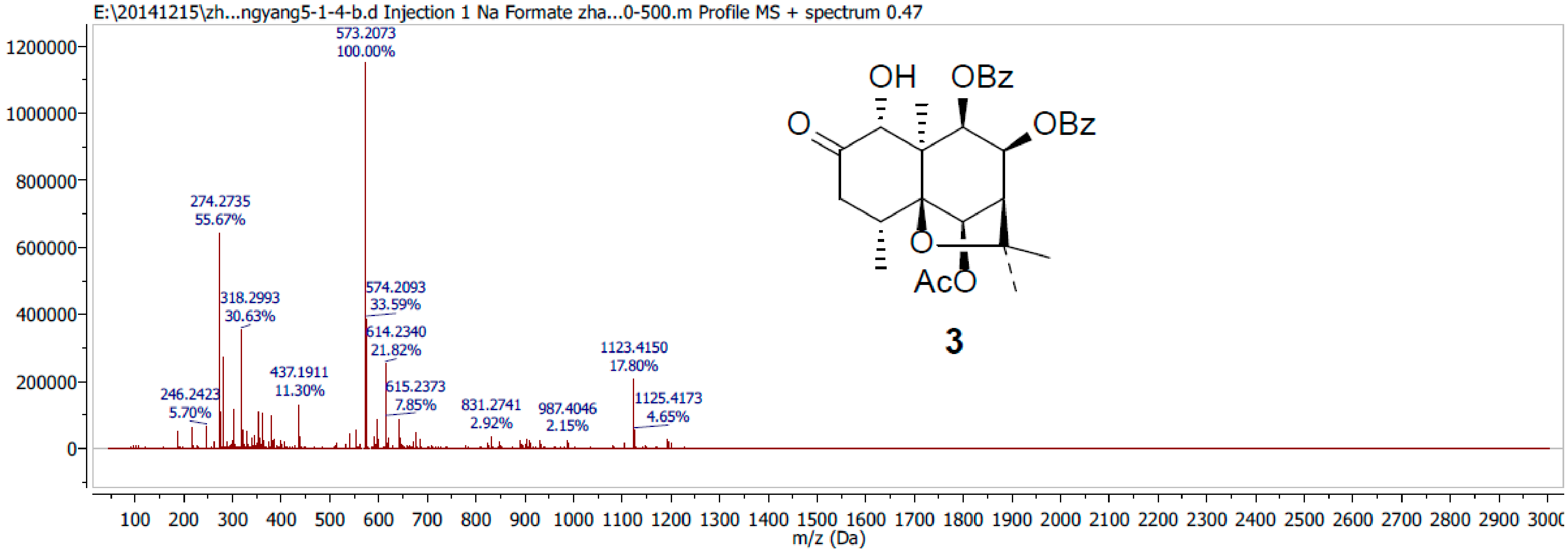

Figure S18. High-resolution mass spectrum of **3**.

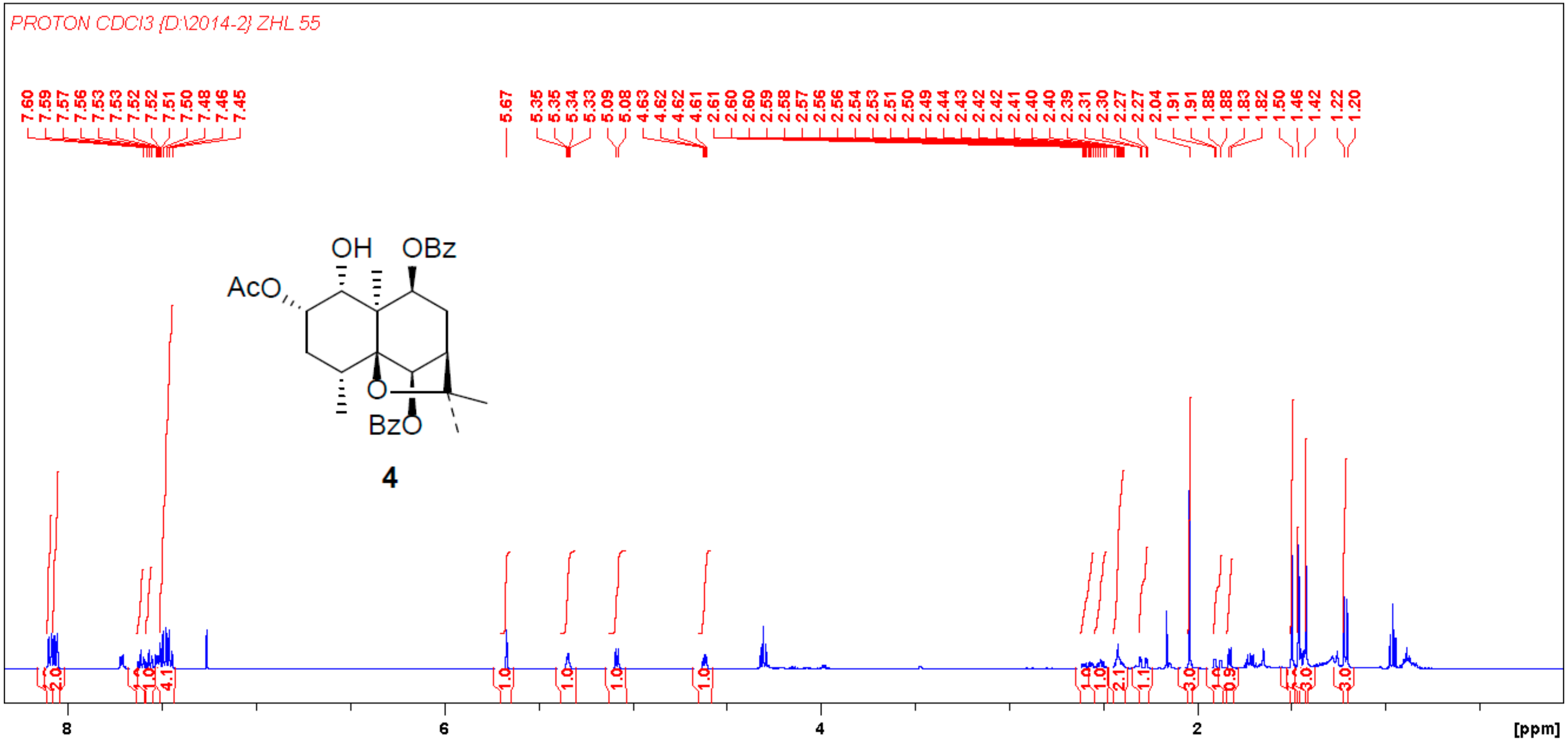

Figure S19. ^{1}H-NMR ($CDCl_3$, 500 MHz) spectrum of **4**.

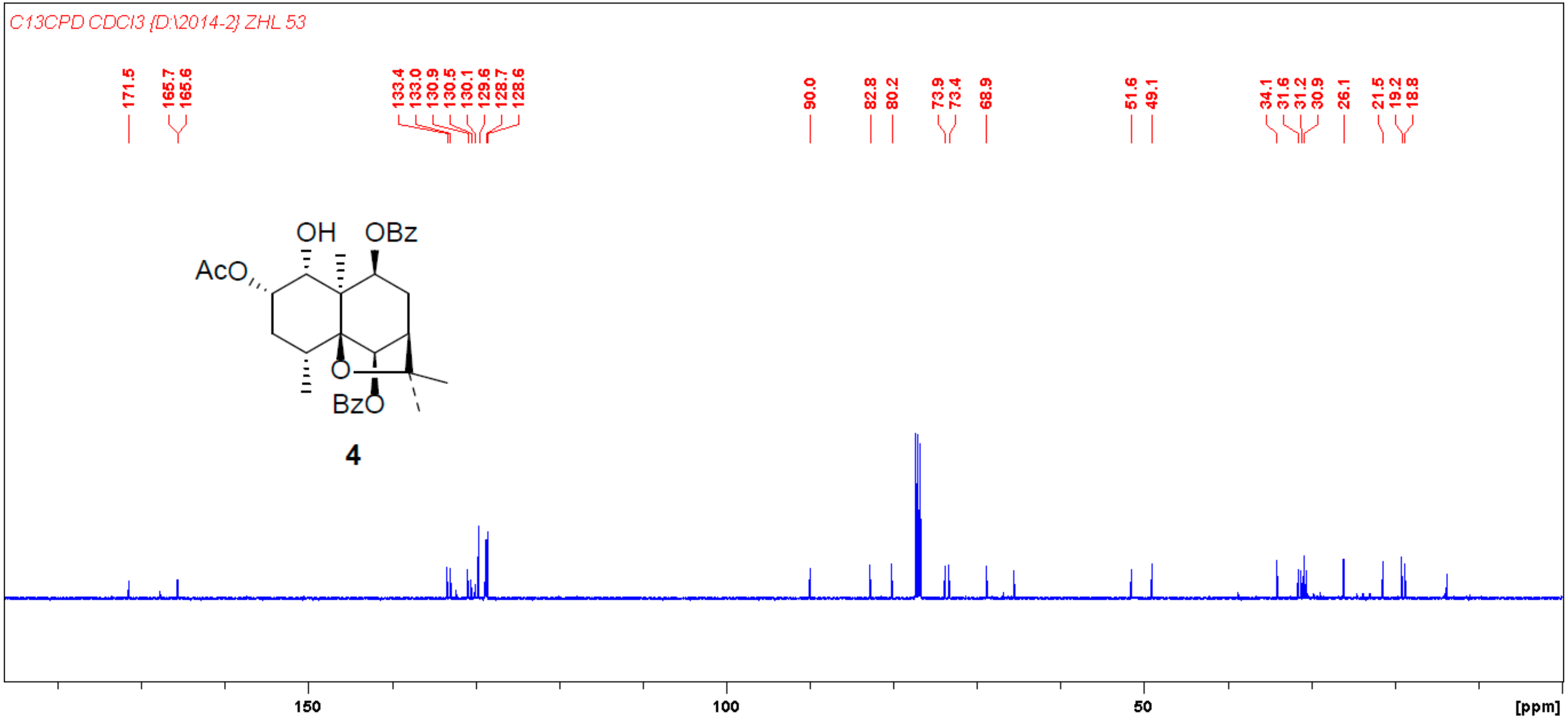

Figure S20. ^{13}C-NMR ($CDCl_3$, 125 MHz) spectrum of **4**.

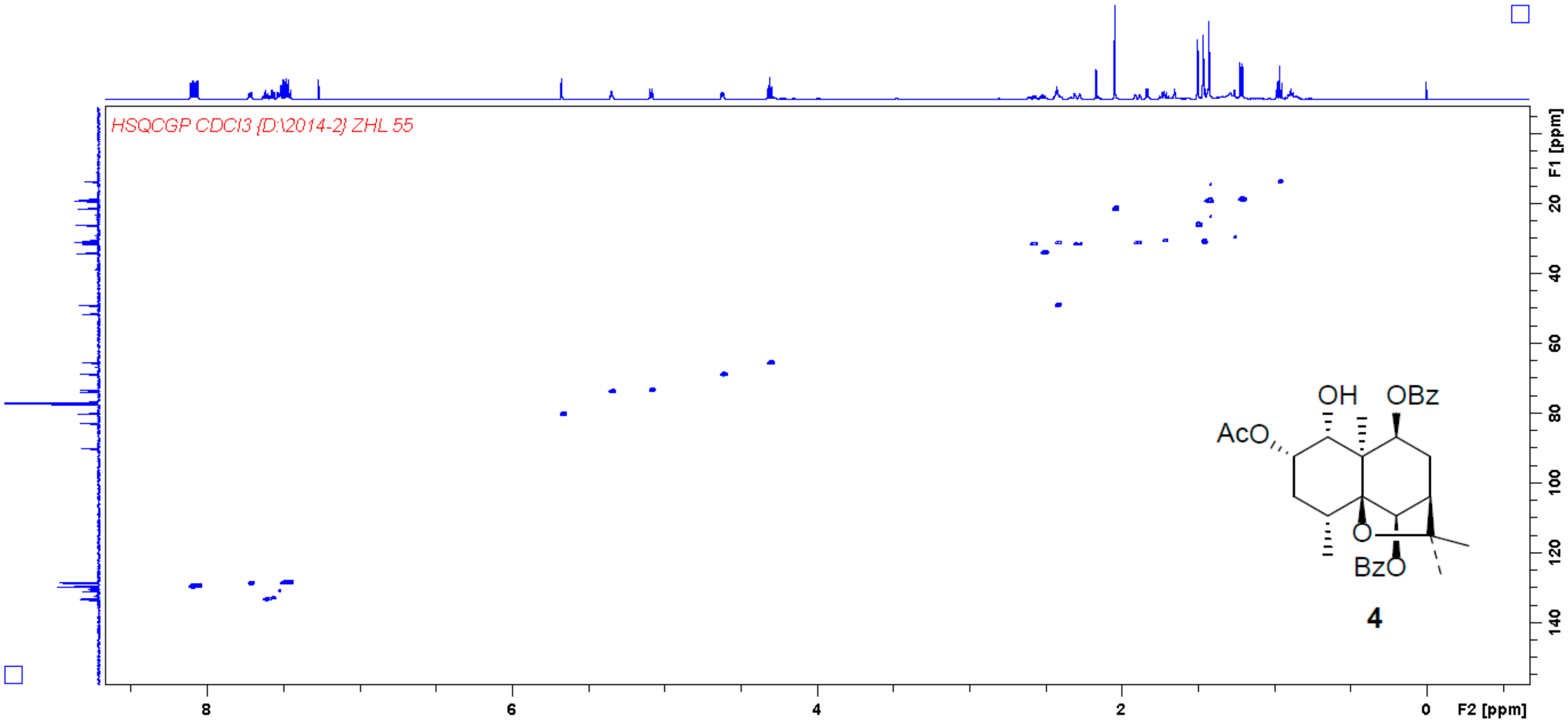

Figure S21. HSQC spectrum of **4**.

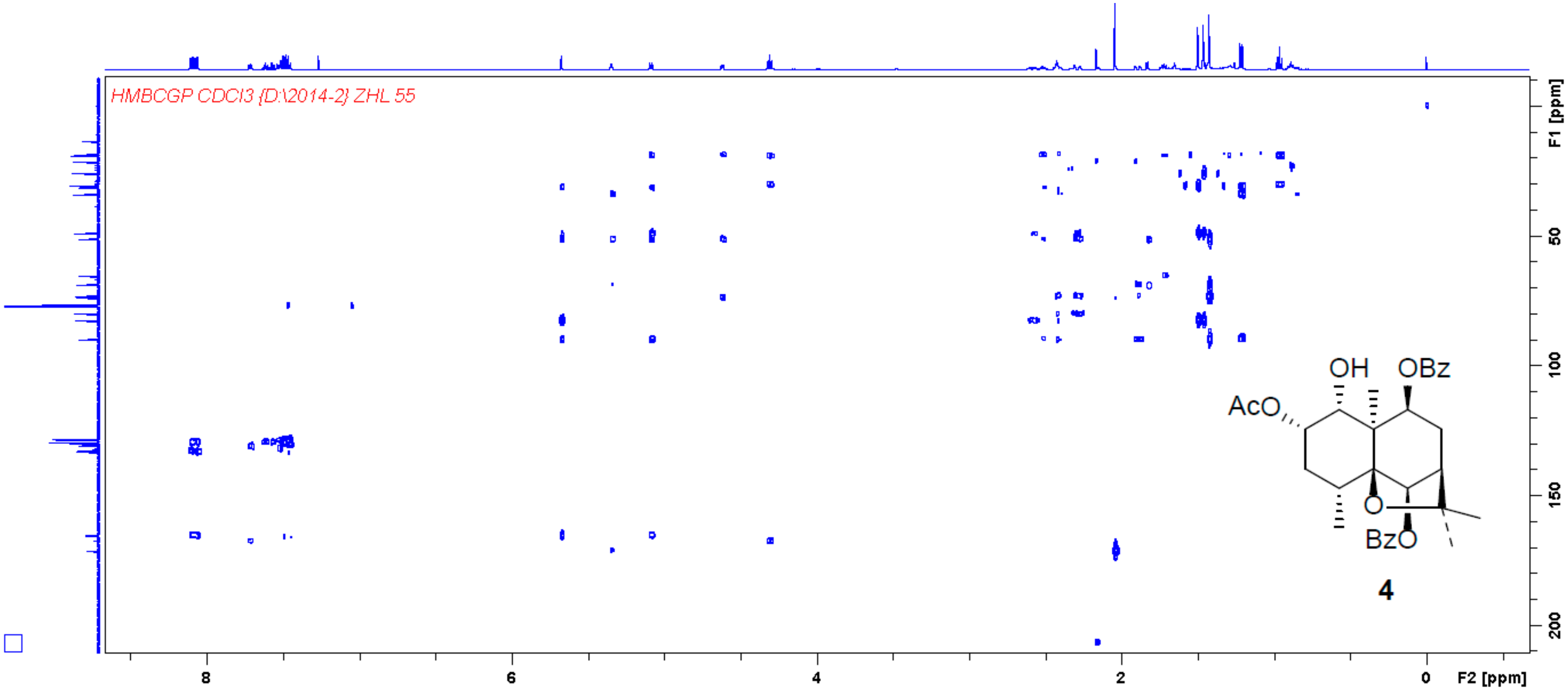

Figure S22. HMBC spectrum of **4**.

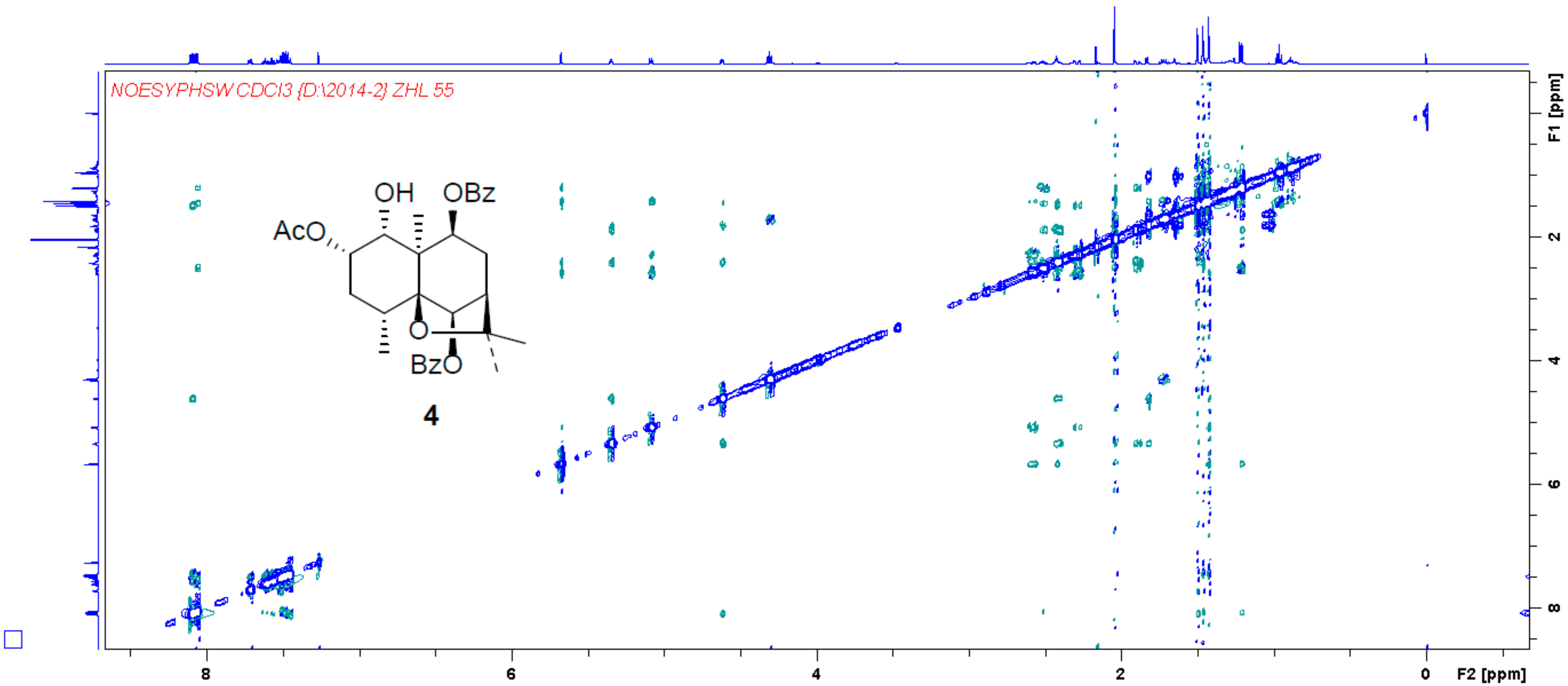

Figure S23. NOESY spectrum of **4**.

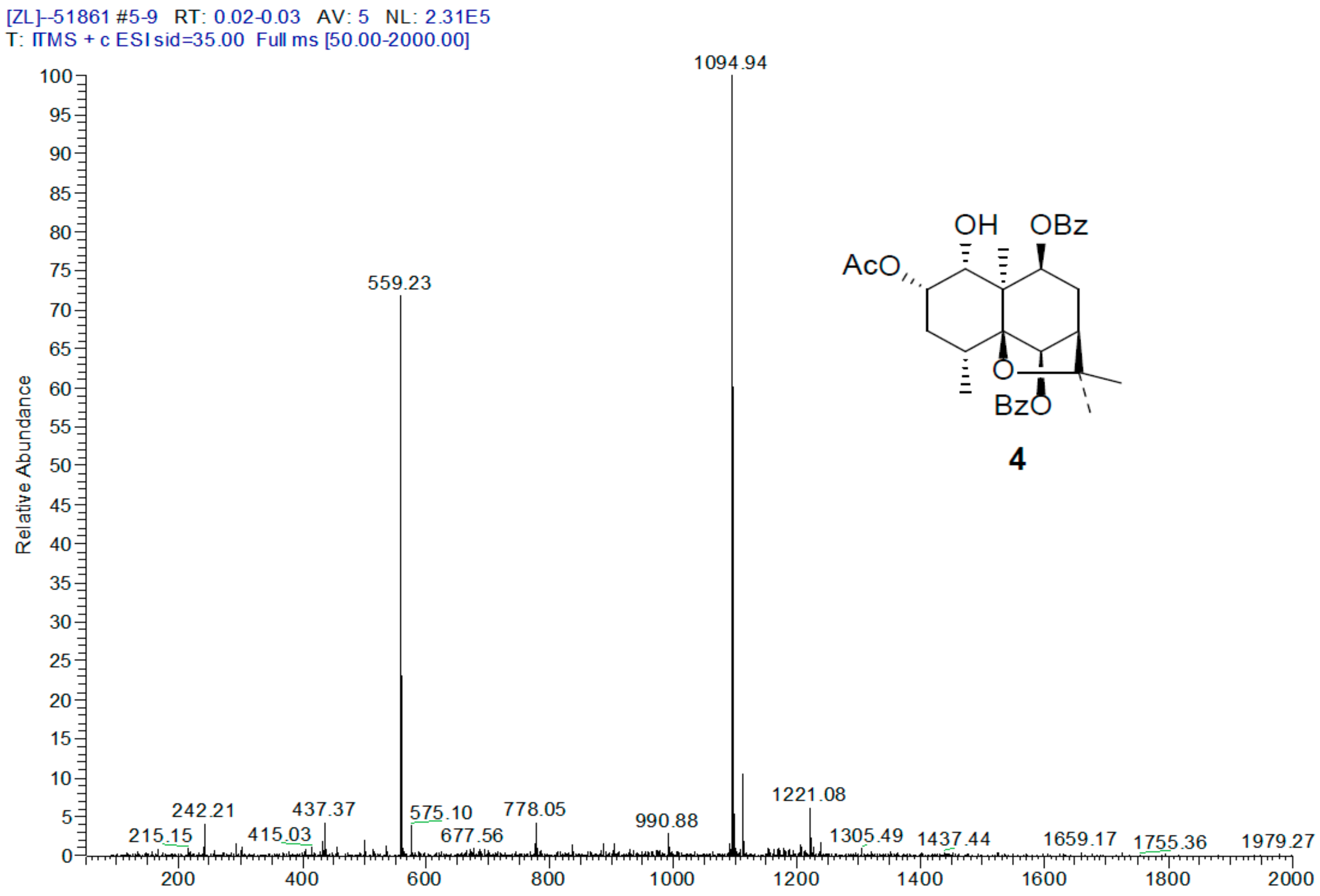

Figure S24. ESI-MS spectrum of **4**.
